# Longitudinal in vivo metabolic labeling reveals tissue-specific mitochondrial proteome turnover rates and proteins selectively altered by parkin deficiency

**DOI:** 10.1038/s41598-023-38484-0

**Published:** 2023-07-14

**Authors:** K. L. Stauch, S. Totusek, A. J. Trease, L. D. Estrella, K. Emanuel, A. Fangmeier, H. S. Fox

**Affiliations:** Department of Neurological Sciences, University of Nebraska Medical Center, Omaha, NE USA

**Keywords:** Molecular neuroscience, Parkinson's disease, Proteomics, Mitochondria

## Abstract

Our study utilizes a longitudinal isotopic metabolic labeling approach in vivo in combination with organelle fraction proteomics to address the role of parkin in mitochondrial protein turnover in mice. The use of metabolic labeling provides a method to quantitatively determine the global changes in protein half-lives whilst simultaneously assessing protein expression. Studying two diverse mitochondrial populations, we demonstrated the median half-life of brain striatal synaptic mitochondrial proteins is significantly greater than that of hepatic mitochondrial proteins (25.7 vs. 3.5 days). Furthermore, loss of parkin resulted in an overall, albeit modest, increase in both mitochondrial protein abundance and half-life. Pathway and functional analysis of our proteomics data identified both known and novel pathways affected by loss of parkin that are consistent with its role in both mitochondrial quality control and neurodegeneration. Our study therefore adds to a growing body of evidence suggesting dependence on parkin is low for basal mitophagy in vivo and provides a foundation for the investigation of novel parkin targets.

## Introduction

Brain function, chiefly synaptic transmission, is a functionally demanding process that leverages the ability of synaptic mitochondria to serve as a regulator of calcium (Ca^2+^) buffering and produce high localized concentrations of adenosine triphosphate (ATP)^1–4^. Mitochondrial quality control is therefore an essential physiological process to maintain robust mitochondrial function to meet bioenergetic and metabolic demands^5–7^. During normal aging, among other mechanisms, the selective autophagic process of mitophagy is a key pathway that mediates the clearance of defective, aged, or damaged mitochondria (or their components)^8–10^. When mitophagy is impaired the accumulation of defective mitochondria leads to ion dysregulation, oxidative stress, and bioenergetic deficits together contributing to synaptic failure and ultimately neuron loss^11–14^. Importantly, impaired mitophagy and the consequent accumulation of defective mitochondria is associated with several human diseases including metabolic syndrome, autoimmune and inflammation disorders, cardiac disease, cancer, and neurodegenerative disorders including Parkinson’s (PD) and Alzheimer’s diseases (AD)^5,6,15–17^.

Mitophagy in general is achieved by the decoration of damaged mitochondria with mitophagy regulating proteins, recruitment and assembly of a phagocytic vesicle that ultimately fuses with the lysosome to achieve bulk protein degradation^5^. Upon loss of mitochondrial membrane potential, two primary mechanisms of mitophagy can occur based on the involvement of the E3-ubiquitin ligase parkin^18^. In parkin-dependent mitophagy, accumulation of the parkin-activator phosphatase and tensin homologue (PTEN)-induced kinase 1 (PINK1) on the outer mitochondrial membrane (OMM), when stimulated by loss of mitochondrial membrane potential, phosphorylates S65 on ubiquitin of monoubiquitinated OMM proteins to recruit parkin and subsequently phosphorylates its ubiquitin-like domain, activating it^19–22^. Once active, parkin then polyubiquitinates several OMM proteins initiating a positive feedback loop with PINK1 culminating in the recruitment of phagocytic machinery and lysosomal degradation^21,23–25^. Several mechanisms of parkin-independent mitophagy have been described, and although they differ in the involvement of various effector proteins, they all lack the involvement of parkin and some lack its activator PINK1^25–30^.

Several studies have demonstrated impaired mitophagy promotes accumulation of dysfunctional mitochondria, impaired synaptic bioenergetics, neuronal loss, elevated reactive oxygen species (ROS), impaired Ca^2+^ handling, and proteostasis^5,6,13,31–33^. These factors in concert with the deposition of Lewy bodies—aggregates of the protein alpha-synuclein—promote inflammation and neuronal death in several neurodegenerative disorders^34–37^. This process is especially detrimental to dopaminergic neurons of the substantia nigra (SN) and their axonal projections to the dorsal striatum, contributing to their destruction, which leads to PD^38–44^. Importantly, loss of function mutations for proteins involved in mitophagy including PINK1, leucine rich repeat kinase 2 (LRRK2), protein deglycase DJ-1 (DJ-1), and parkin result in early onset familial forms of PD^45^. Additionally, mutations in mitophagic factors have been associated with autoimmune disorders (LRRK2)^46^ and tumorigenesis (parkin)^47^. In the laboratory, genetic ablation of one or more of the genes encoding these proteins has been used to model the onset and progression of PD in mice and rats, although none result in a true phenocopy of the human disease^48^. Notably, a commonality observed in several of these models is altered mitochondrial bioenergetics and homeostasis^49–51^.

While mitophagy results in the turnover of the damaged mitochondrion little is known about basal turnover of mitochondrial proteins at the synapse nor the importance of parkin to basal turnover in vivo in mammalian models, although some subtle evidence exists in invertebrate models^23,52–54^. With the importance of mitochondrial protein quality control, proteostasis, and mitochondrial dysfunction in neurodegenerative disorders such as PD, there exists a substantial need to better understand these processes.

In the current study we sought to investigate the consequences of parkin insufficiency (parkin knock-out) on the mitochondrial proteome in vivo. Using genetic mouse models and mass spectrometry coupled with longitudinal isotopic labeling we assessed the basal levels of mitochondrial protein turnover in mitochondria isolated from liver tissue or synaptic terminals from 3-month-old wild-type (WT) or parkin knock-out (PKO) mice. Our hypothesis was that loss of parkin, and thus disruption of the parkin-PINK1 mitophagy pathway, would lead to increased half-lives of mitochondrial proteins. Our study revealed differential basal turnover rates of hepatic and synaptic mitochondrial proteins—specifically, we report the median half-life of the synaptic mitochondrial proteome is substantially longer than that of hepatic mitochondria. Further we find differential effects of loss of parkin. Notably, there was a general modest increase in mitochondrial protein expression in mice lacking parkin compared to WT that correlated with reduced turnover (increased protein half-life) in liver. However, in brain striatal synaptic mitochondria, the effect was less pronounced, leading us to conclude that parkin-dependent mechanisms do not contribute to homeostatic regulation of mitochondria in the examined areas.

## Materials and methods

### Study design

In the study presented herein wild-type (WT, Stock No: 000664, C57BL/6J) and parkin knock-out (PKO, Stock No: 006582, B6.129S4-*Prkn*^*tm1Shn*^/J)^55^ mice were obtained from The Jackson Laboratory. The mice were maintained on a 12 h light/dark cycle in a temperature-controlled environment with free access to standard mouse chow and water. Experimental mice were bred in-house in accordance with institutionally approved breeding protocols. Mice used in this study were weighed at 30, 60, and 90 days of age. PKO mice weighed significantly less than WT mice at all ages (Supplementary Fig. S1). For metabolic labeling (see below) 12–15 male mice of either strain were randomly assigned to groups based on days of labeled diet (see section below), in total 48–60 mice of each strain were used in the study. Liver mitochondria (n = 6) were isolated at random from 6 individual mice from each time point cohort, while striatal synaptic mitochondria samples (n = 3) were derived from randomly pooling isolated mitochondria from 4–5 mice of each time point cohort. All breeding and experimental procedures described herein were approved by The University of Nebraska Medical Center Institutional Animal Care and Use Committee (IACUC; protocol numbers 10–043 [experimental] and 08–037 [breeding]) and carried out in accordance with approved protocols and regulations. Sample sizes were chosen based on the minimum number of animals necessary to yield sufficient striatal synaptic mitochondria for downstream proteomics analysis while maintaining a minimum of three biological replicates. Study methods within were conducted and are reported in accordance with the ARRIVE guidelines.

### Stable isotope labeling

At 52 days of age, all mice were started on a synthetic diet (Teklad TD.01084, Envigo) to facilitate the subsequent substitution of a heavy-labeled leucine diet to enable protein turnover measurements. After 21 days, the mice were randomly assigned to 4 groups: 3-, 7-, 12-, or 17-days stable isotope labeling. The mice (16 mice per group) were started on a leucine-deficient synthetic diet (Teklad TD.170067, Envigo) with the light leucine fully replaced by 11.1 g/kg of deuterated [5,5,5-^2^H_3_]-L-leucine (Cambridge Isotope Laboratory) at 87, 83, 78, or 73 days of age. At 90 days of age, all mice were euthanized by cervical dislocation at 4 time points: 3-, 7-, 12-, and 17-days after they were switched to the [^2^H_3_]-leucine diet. The WT mice gained, on average, ~ 40.8% of their starting weight between 30 and 60 days of age (Supplementary Fig. S1). Between 30 and 60 days of age, the PKO mice gained, on average, ~ 58.3%. On average, the WT and PKO mice gained ~ 17.8% and ~ 23.2% between 60 and 90 days of age, respectively.

### Isolation of liver mitochondria

Hepatic mitochondria were isolated as previously described^56^. Liver tissue was homogenized in ice-cold mitochondrial isolation buffer (MSHE + BSA): 70 mM sucrose, 210 mM mannitol, 5 mM HEPES, 1 mM EGTA, and 0.5% (w/v) fatty acid free (FAF)-BSA (pH 7.2) using 10 strokes in a Dounce homogenizer. The homogenate was centrifuged for 10 min at 600×*g*, the supernatant was collected and centrifuged for 10 min at 15,000×*g*. The pellet was resuspended in MSHE + BSA and layered on top of a 26% and 60% Percoll gradient (prepared from 100% Percoll solution containing 70 mM sucrose, 210 mM mannitol, 5 mM HEPES, 1 mM EGTA, and 0.5% (w/v) FAF-BSA (pH 7.2)). Following centrifugation for 30 min at 34,750×*g* (SW40-Ti), the banding near the interface of the 26% and 60% gradient layers, containing mitochondria, was collected and diluted in MSHE + BSA. The mitochondrial suspension was centrifuged for 10 min at 16,700×*g*. The pellet was resuspended in mitochondrial assay solution (MAS, 1x): 70 mM sucrose, 220 mM mannitol, 10 mM KH_2_PO_4_, 5 mM MgCl_2_, 2 mM HEPES, 1 mM EGTA, and 0.2% (w/v) FAF-BSA (pH 7.2) and centrifuged for 10 min at 13,000×*g*. The final liver mitochondrial pellet was lysed in 100 mM Tris–HCl with 4% (w/v) SDS and 0.1 M DTT adjusted to pH 7.6 using brief sonication and incubation at 95 °C for 5 min. The Pierce 660 nm Protein Assay was used for protein quantification.

### Isolation of striatal synaptic mitochondria

Synaptic mitochondria were isolated using our previously published method^57^. Briefly, the striata, dissected from 3–4 mice, were pooled and homogenized in ice-cold MSHE + BSA using 10 strokes in a Dounce homogenizer. The homogenate was centrifuged for 3 min at 1300×*g*, the supernatant was collected, the pellet was resuspended in MSHE + BSA and the centrifugation step was repeated. The supernatants were pooled and centrifuged for 10 min at 21,000×*g*, and the resulting pellet was resuspended in 15% Percoll and layered on top of a 24% and 40% Percoll gradient (prepared from 100% Percoll solution). Following ultracentrifugation for 10 min at 30,700×*g*, the banding near the interface of the upper two gradient layers, containing synaptosomes, was collected and diluted in MSHE + BSA.

To isolate synaptic mitochondria, the synaptosomal fraction was transferred to a nitrogen cavitation vessel and the pressure was equilibrated to 900 psi for 15 min followed by depressurization to atmospheric pressure to release the synaptic mitochondria. The synaptic mitochondria suspension was added to the top of 24% Percoll and ultracentrifuged for 10 min at 30,700×*g*. The synaptic mitochondria pellet was resuspended in MSHE + BSA and centrifuged for 10 min at 8000×*g*. The pellet was washed twice with MAS and centrifuged for 10 min at 8000×*g*. The synaptic mitochondria pellet was lysed in 100 mM Tris–HCl with 4% (w/v) SDS and 0.1 M DTT adjusted to pH 7.6 using brief sonication and incubation at 95 °C for 5 min. The Pierce 660 nm Protein Assay was used for protein quantification.

### Protein digestion

Mouse liver and striatal synaptic mitochondrial protein sample lysates were digested with trypsin using the filter-aided sample preparation (FASP) method^58^. Oasis mixed-mode weak cation exchange cartridges were used to desalt the resultant peptides prior to dehydration with a Savant ISS 110 SpeedVac concentrator. Dehydrated peptides were resuspended in 0.1% formic acid prior to quantification using a NanoDrop 2000 UV–vis Spectrophotometer in conjunction with the Scopes^59^ method for peptide quantification by absorbance at 205 nm.

### Mass spectrometry acquisition

#### Untargeted proteomics of liver mitochondria

##### Liquid chromatography

Peptides were cleaned with PepClean C18 spin columns, re-suspended in 2% acetonitrile (ACN) and 0.1% formic acid (FA), and 2 µg of each sample was loaded onto a trap column Acclaim PepMap 100 75 µm × 2 cm C18 LC Columns at a flow rate of 4 µl/min followed by separation with a Thermo RSLC Ultimate 3000 on a Thermo Easy-Spray PepMap RSLC C18 75 µm × 50 cm C-18 2 mm column utilizing a step gradient of 9–25% solvent B (0.1% FA in 80% ACN) from 10 to 100 min and 25–45% solvent B for 100–130 min at 300 nL/min and 50 °C with a 155 min total run time.

##### Mass spectrometry

Eluted peptides were analyzed by a Thermo Orbitrap Fusion Lumos Tribrid mass spectrometer in data dependent acquisition (DDA) mode. Full-scan survey mass spectra were acquired in the Orbitrap at a resolution of 60,000 from 375 to 1500 m/z. The AGC target for MS1 was set at 100% and ion filling time at 50 ms. The most intense ions with charge state 2 thru 5, isolated in 3 s cycles and fragmented using HCD fragmentation at 30% normalized collision energy, were detected at a mass resolution of 15,000. The AGC target for MS2 was set at 100% and ion filling time at 22 ms with a dynamic exclusion of 15 s and a 10 ppm mass window.

#### Untargeted proteomics of striatal synaptic mitochondria

##### Liquid chromatography

Synaptic mitochondrial peptides were sent to Bioproximity and mass spectrometry was performed on a Q-Exactive HF-X Quadrupole Orbitrap instrument coupled with an EASY nLC 1200.

##### Mass spectrometry

Eluted peptides were analyzed by a Q-Exactive HF-X Quadrupole Orbitrap mass spectrometer in DDA mode. Full-scan survey mass spectra were acquired in the Orbitrap at a resolution of 60,000 from 350 to 1400 m/z. The AGC target for MS1 was set at 1e5 and ion filling time at 45 ms. The most intense ions with charge state 2 thru 5, isolated in 1.3 s cycles and fragmented using HCD fragmentation at 27% normalized collision energy, were detected at a mass resolution of 15,000. The AGC target for MS2 was set at 4.50e3 and ion filling time at 22 ms with a dynamic exclusion of 30 s and a 10 ppm mass window.

### Mass spectrometry analysis (Abundance)

Liver (n = 24; Lumos Tribrid—see Mass Spectrometry Acquisition section) or synaptic mitochondria (n = 12; Q-Exactive—see Mass Spectrometry Acquisition section) from 3-month-old WT and PKO male mice were isolated, lysed and quantified by Pierce 660 nm protein assay. Fifty (50) µg of protein was prepared for mass spectroscopy using the FASP method as previously described^57,58^. Two (2) µg of eluted peptide from each was injected for analysis as described above. Raw files were searched using MaxQuant (2.0.3.0)^60,61^ against the Uniprot *Mus musculus* proteome (UP000000589) using the MaxQuant^60,61^ internal contaminant list to obtain protein identifications. Enzymatic cleavage specificity was set to trypsin with a maximum of two missed cleavages. Label multiplicity was set to two with a heavy label to account for tri-deuterated leucine (+ 3.0188325 Da). Variable modifications were set to oxidation (M), phospho (STY) and acetyl (N-term) and the only fixed modification used was carbamidomethyl C. Quantitation was achieved using the MaxQuant^60,61^ standard label-free quantification algorithm with default settings plus match between runs, second peptide and dependent peptide search with an FDR of 0.01. Total LFQ intensity for each protein ID in each sample of the proteinGroups.txt file was determined by summing of light and heavy LFQ-intensity values prior to subsequent analysis. Using LFQ-Analyst^62^, the corrected raw data files were normalized based on the assumption that the majority of proteins do not change between the different conditions. Statistical analysis was performed using an in-house generated R script based on the proteinGroups.txt file. Contaminants, reverse hits, and proteins identified “only by site” were filtered out. Additionally, proteins identified by a single peptide and those not identified/quantified consistently in same experimental condition were also removed. The LFQ data was converted to log_2_ scale, samples were grouped by conditions and missing values were imputed using the ‘Missing not At Random’ (MNAR) method, employing random sampling of a left-shifted Gaussian distribution of 1.8 StDev apart with a width of 0.3. Protein-wise linear models combined with empirical Bayes statistics were used for the differential expression analyses. The density of initial search results is illustrated in Supplementary Fig. 2. A list of DE proteins for each comparison was generated by the R Bioconductor package, limma^63^. An adjusted p-value cutoff of 0.05 (Benjamini–Hochberg method) and an absolute log_2_ fold change of 1 were set to identify significant DE proteins in each pairwise comparison. Proteins deemed significant by this method were further subjected to a missing value cutoff, wherein only proteins that were quantified in a minimum of 50% of samples from each genotype (i.e. in hepatic and synaptic mitochondria 12 and 6 valid values, respectively, were required in each WT and PKO). Differentially expressed proteins that were quantified in both tissues were hierarchically clustered by Euclidean distances with average linkage in Perseus (1.6.15.0)^64^ with 21 row clusters and all other default settings. These clustered proteins were then filtered for those that were deemed significant in one or both tissues by quantitative analysis.

### Mass spectrometry analysis (Turnover)

Precursor feature refinement of MS data for turnover analysis was performed using Hardklor (v2.3.0)^65,66^ and Bullseye (v1.30)^67^. A database search was performed using Comet (2018.01 rev. 2)^68^ against a UniProt library composed of canonical proteins for *Mus musculus* (UP000000589) in addition to contaminant proteins. A static modification of 57.021461 Da for cysteine was set to account for carbamidomethyl modifications, while dynamic modifications of 3.0188325 Da for leucine and 15.9949 Da for methionine were set to account for [5,5,5-^2^H_3_]-leucine and methionine oxidation, respectively. The precursor mass tolerance was set to 10 ppm with tryptic enzyme specificity allowing two missed cleavages per peptide. False discovery metrics were re-scored with the Crux-Percolator (3.02.0)^69,70^ using reversed sequences as decoys. Proteins were filtered with an FDR q-value threshold of 0.01, and the output was transformed to the BlibBuild^71^ spectrum sequence list format for import into Topograph^72^ using a custom R script.

Protein half-lives were calculated based on data from all peptides detected using the Topograph-daily x64 release. Proteins were represented by at least 10 total values of percent newly synthesized per genotype. Shared peptides were excluded from the analysis and only unique peptides were used. Cutoffs were applied utilizing the quality controls generated by Topograph; turnover score ≥ 0.98, deconvolution score ≥ 0.95, total area under the curve ≥ 1,000,000, and data points above or below the protein mean by more than 2 standard deviations. For the half-life calculations, data points from all biological replicates were pooled. Proteins with excessive variability of percent newly synthesized values were excluded and determined as follows. To create a measure analogous to a coefficient of variation, the 95% confidence interval generated by Topograph for each half-life was divided by the half-life value. Proteins with a 95% confidence interval/half-life ratio ≥ 0.3 were excluded from the analysis^23^. This data censor resulted in a total of 742 and 412 proteins with quantified half-lives for comparison between WT and PKO mice in hepatic and striatal synaptic mitochondria, respectively (Supplementary Fig. 3).

### Pathway analysis

Log_2_ fold change values for all identified proteins shared by both liver and synaptic mitochondria were analyzed with the use of QIAGEN IPA (QIAGEN Inc.)^73^ to identify pathways affected by parkin insufficiency. Summary networks were created for each tissue type alone, and a comparison analysis was employed to identify predicted activation of canonical pathways. Canonical pathway activation z-scores were subjected to hierarchical clustering by Euclidean distances with average linkage using Perseus (1.6.15.0)^64^, with 10 row clusters and all other default settings.

### Western blot analysis

Liver mitochondria were isolated as above and lysed using 100 mM Tris–HCl with 4% (w/v) SDS and 0.1 M DTT adjusted to pH 7.6 by sonication and incubation at 95 °C for 5 min. Protein concentration was determined using the Pierce 660 nm Protein Assay. Fifteen (15) µg of total protein was resolved on either (Nnt, ubiquitin) 4–12% Bis–Tris NuPAGE Bolt gels using a MOPS/SDS buffer system or (NdufV2) 15% Tris gels using the Tris/Glycine buffer system. Gels were transferred to poly-vinylidene-fluoride membrane using the iBlot dry transfer system. Membranes were blocked for 1 h at room temp with 3% non-fat milk in tris-buffered saline with 0.1% Tween-20 (TBST). Membranes were incubated overnight with primary antibodies diluted in 1% non-fat milk in TBST (Nnt 1:1000, Sigma Aldrich [HPA004829]; NdufV2 1:750, Sigma Aldrich [HPA004829]; total ubiquitin 1:500, Invitrogen [13–1600]). Membranes were washed 3 × 10 min with TBST and then incubated with appropriate near-infrared conjugate secondary antibodies for 1 h at room temp (goat anti-mouse-680, goat anti-rabbit-800 1:20,000 each, Licor). After 3 additional 10 min washes with TBST membranes were imaged on a Licor Odyssey at 2 min per channel and quantified by densitometry. Expression of target protein was normalized to total protein loading via Coomassie staining and statistical analysis was performed in GraphPad Prism 9. Raw uncropped blot and Coomassie images have been provided in Supplementary Fig. 4.

## Ethical approval

The animal study was reviewed and approved by the University of Nebraska Medical Center Institutional Animal Care and Use Committee.

## Results

### Loss of parkin alters expression of the mitochondrial proteomes of liver and synapses

**Figure 1:**
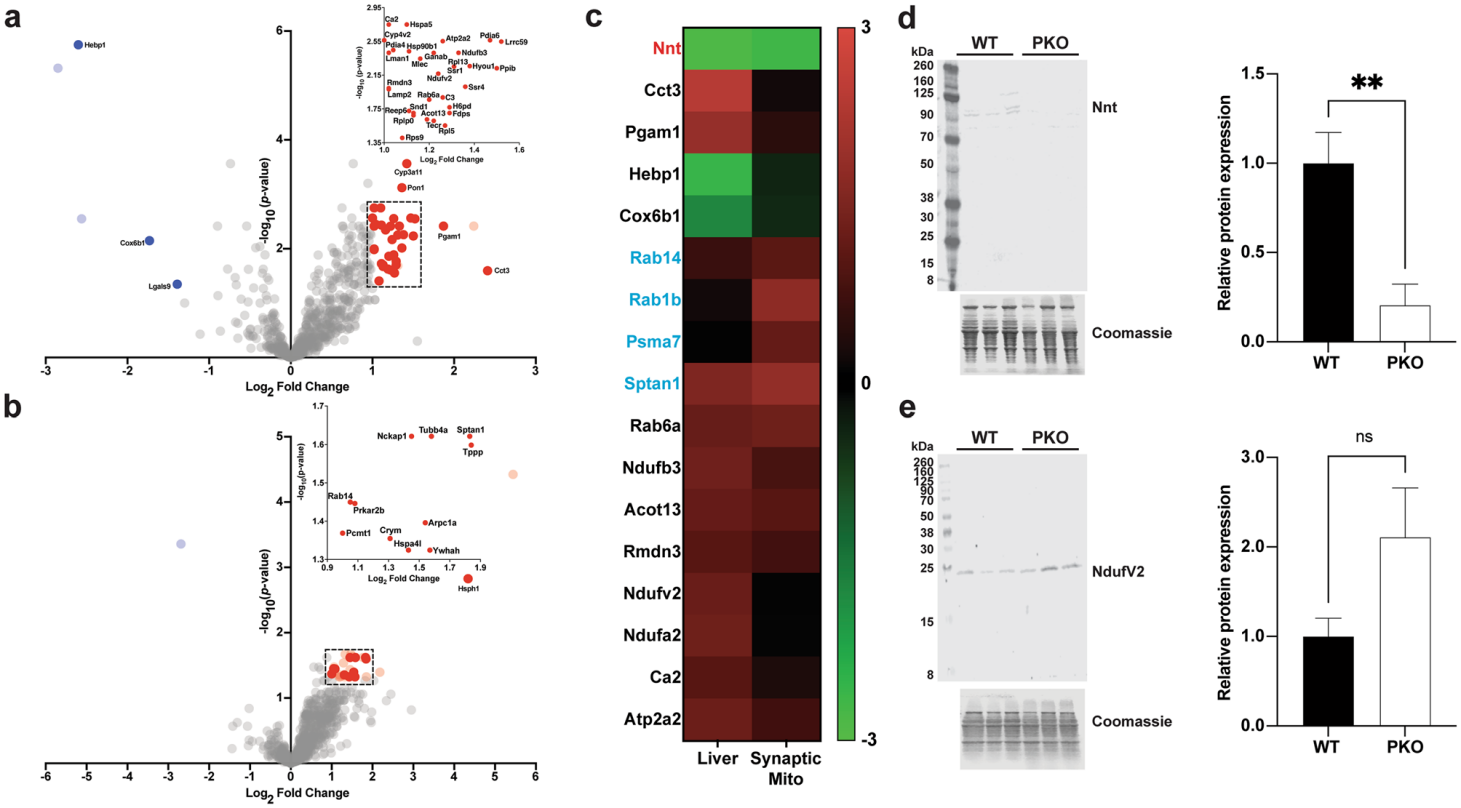
Differential expression of mitochondrial proteins induced by loss of parkin. Label-free quantitative proteomics were used to determine the effects of loss of parkin on the mitochondrial proteomes of (**a**) liver and (**b**) synaptic mitochondria. Proteins considered significantly up- (dark red) or down-regulated (dark blue) were determined by a log_2_ fold change >|1| in PKO vs WT, adjusted *p* value < 0.05, and were quantifiable in no less than half of the samples in each genotype. Lighter colors indicate those proteins that had were quantifiable in less than half of the samples in each genotype. (**c**) Heatmap of DE proteins shared by liver and synaptic mitochondria and were deemed significant in liver mitochondria (black text), synaptic mitochondria (blue text), or both tissues (red text). Heatmap was generated in GraphPad Prism 9 using filtered LFQ-analyst^62^ log_2_ fold change outputs after hierarchical Euclidean clustering in Perseus (1.6.15.0)^64^. Western blot and densitometric quantification of (**d**) Nnt and (**e**) NdufV2 from liver mitochondrial isolates. Target protein expression was normalized as a function of total protein. Statistical significance was determined by one-tailed unpaired student’s t-test.

First, to assess if loss of parkin alters the abundance of mitochondrial proteins, we assessed the proteome of mitochondria from liver and brain, two organs known to show high expression of parkin^74,75^. For the brain, to increase the relevance of our work to Parkinson’s disease (PD), synaptic mitochondria were isolated from the striatum, the target of projections from the SN. Expression profiles of the proteomes of mitochondria isolated from liver tissue and striatal synapses were determined by quantitative label-free proteomics. We reproducibly quantified 809 and 847 proteins in total from hepatic and synaptic mitochondrial preparations, respectively. Using an expression absolute log_2_ fold change threshold of 1.0 and an adjusted *p* value cutoff of ≤ 0.05 resulted in 42 differentially expressed (DE) proteins in parkin knock-out (PKO) relative to wild-type (WT) in hepatic (4 down- and 38 up-regulated; Fig. 1a—blue and red dots, respectively), and 27 in synaptic mitochondria (1 down- and 26 up-regulated; Fig. 1b—blue and red dots, respectively). Complete lists of quantified proteins are provided in Supplementary Table S1 (liver mitochondria) and S2 (striatal synaptic mitochondria). Further quality control was implemented to enhance confidence by restricting the number of missing values per protein to ≤ 50% of each genotype (i.e. ≤ 12 and 6 missing values per genotype from liver and synaptic mitochondria, respectively) ultimately reducing the number of significant DE proteins to 38 (2 down- and 36 up-regulated; Fig. 1a—dark blue and dark red dots, respectively) in hepatic mitochondria, and 12, all upregulated (Fig. 1b—dark red dots), in synaptic mitochondria. Notably 330 proteins were shared between tissues of which 17 were determined as significantly DE (disregarding the missing value threshold) in one or both mitochondrial populations (Fig. 1c—black text liver only, blue text synaptic only, red both). Interestingly, the only DE protein that was significant in both hepatic and synaptic mitochondria was NAD(P) transhydrogenase (Nnt) and it was down-regulated by loss of parkin in either tissue. The PKO log_2_ fold change in Nnt abundances relative to WT were − 2.85 (*p* = 4.81E−6) and − 2.69 (*p* = 5.48E−6) in hepatic and striatal synaptic preps, respectively. In general, these 17 proteins displayed similar expression directionality is both tissues. In validation of our MS1-based proteomics quantification, we immunoblotted for Nnt and NdufV2 expression in liver mitochondrial isolates (Fig. 1d,e). In support of our proteomics results expression was decreased and increased, for Nnt and NdufV2, respectively in PKO liver mitochondria compared to WT liver mitochondria. NdfuV2, not reaching significance, likely due to decreased sensitivity of immunoblot compared to LC–MS/MS.

### Pathway analysis revealed global impact of parkin insufficiency

**Figure 2:**
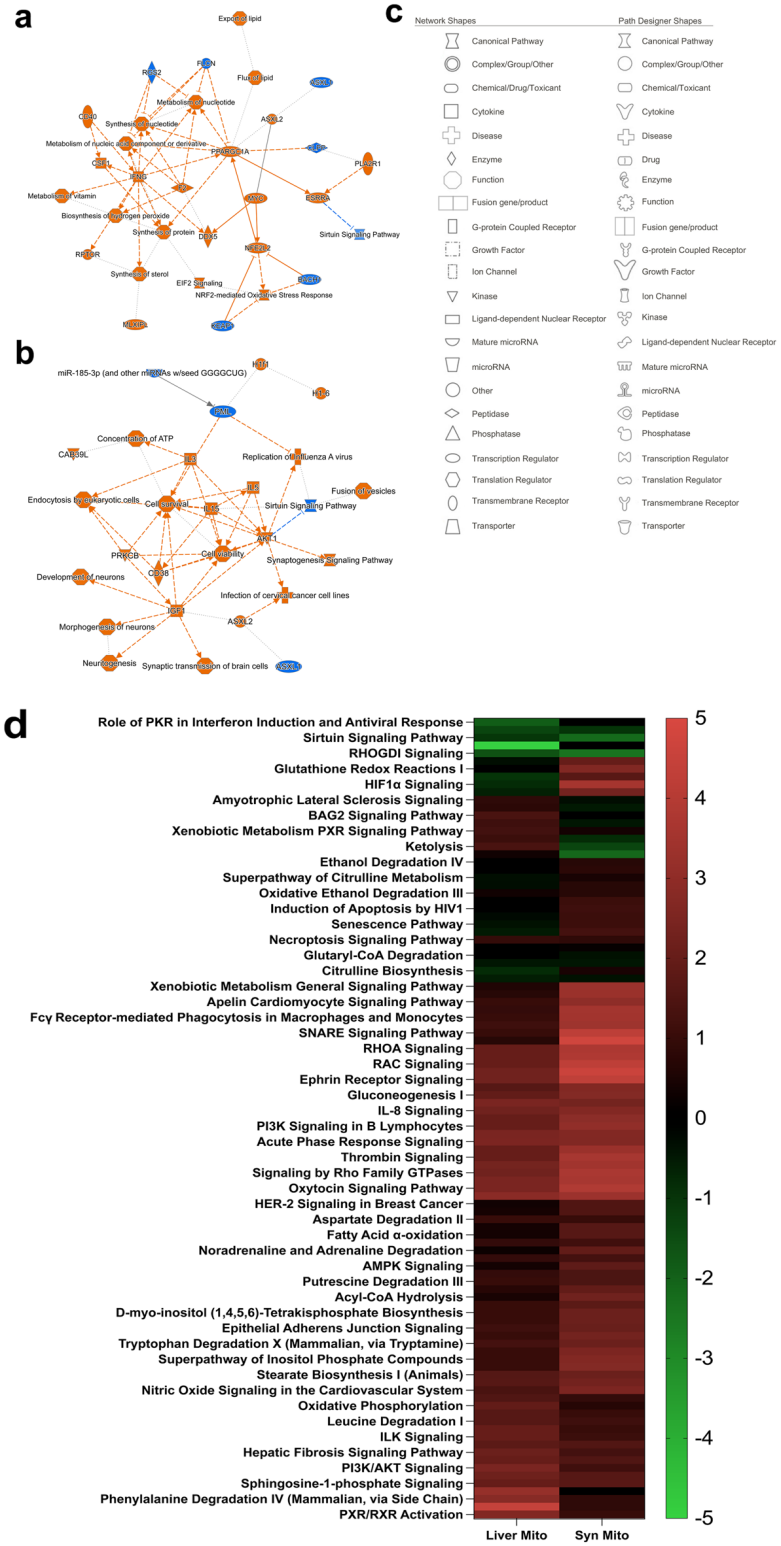
Pathway analysis reveals differential effects of parkin deficiency in liver and synaptic mitochondria. Ingenuity pathway analysis (IPA) was used to predict global changes in relationships between individual elements in the proteomes of (**a**) liver and (**b**) synaptic mitochondria, including upstream regulators, interactors, and signaling effectors. Activation z-scores as predicted by IPA are represented as orange (positive) and blue (negative). (**c**) The IPA legend for network symbols in (**a**–**b**) is provided. Images in a-c were modified from IPA outputs. **(d)** Comparison of canonical pathways affected by parkin deficiency in liver and synaptic mitochondria as predicted by IPA. Heatmap was generated in GraphPad Prism 9 from activation scores generated by IPA after Euclidean hierarchical clustering in Perseus (1.6.15.0)^64^.

To uncover similarities and differences in the global effects on mitochondrial protein abundance and impact on canonical pathways by parkin insufficiency, Ingenuity pathway analysis^73^ (IPA) was used to analyze the quantified proteins in common to both liver and striatal. Graphical summaries of the global IPA analysis (i.e., Canonical Pathways, Upstream Regulators, Transcriptional Regulators, etc.) for hepatic and synaptic mitochondria are presented in Fig. 2a,b, respectively. Notably, in hepatic mitochondria the central node with positive activation was peroxisome proliferator activated receptor γ (Ppargc1) coupled with an overall state of activation of pathways and regulators related to mitochondrial biogenesis and liver detoxification (Fig. 2a, Supplementary Table S3). Interestingly, the sirtuin signaling pathway, which plays a role in cell survival and stress response, was determined to have a negative activation score (Fig. 2a). In synaptic mitochondria the primary central nodes, exhibiting positive activation, were “Cell survival” and “Cell viability” (Fig. 2b, Supplementary Table S4). Sirtuin signaling was also predicted and similar to hepatic mitochondria, the activation score was also negative but to a greater degree. Unlike liver mitochondria pathways related to mitochondrial biogenesis are less prevalent in striatal synaptic mitochondria when parkin is lost. A legend for symbols used in the IPA outputs is provided in Fig. 2c. To make a direct comparison between the hepatic and synaptic mitochondria, a comparison analysis was conducted using IPA and the activation z-scores of predicted “Canonical Pathways” are reported in the heatmap in Fig. 2d and the complete list is available in Supplementary Table S5 (canonical pathways are well-defined biochemical cascades in the cell that transduce a specific functional biological consequence). Although largely similar, hepatic and synaptic mitochondria differ in the Rho GDP-dissociation inhibitor (Arhgdia) signaling (positive activation in synaptic mitochondria) and pathways related to the TCA cycle, ketogenesis, ketolysis, as well as amyotrophic lateral sclerosis response (negative activation in synaptic mitochondria; Fig. 2d). The majority of the canonical pathways displaying positive activation scores in both hepatic and synaptic mitochondria are related to energy amino acid metabolism, cholesterol metabolism, and importantly, pathways of neurotransmitter degradation.

### Proteins associated with ubiquitin-like conjugation processes are altered in hepatic mitochondria when parkin is lost

**Figure 3:**
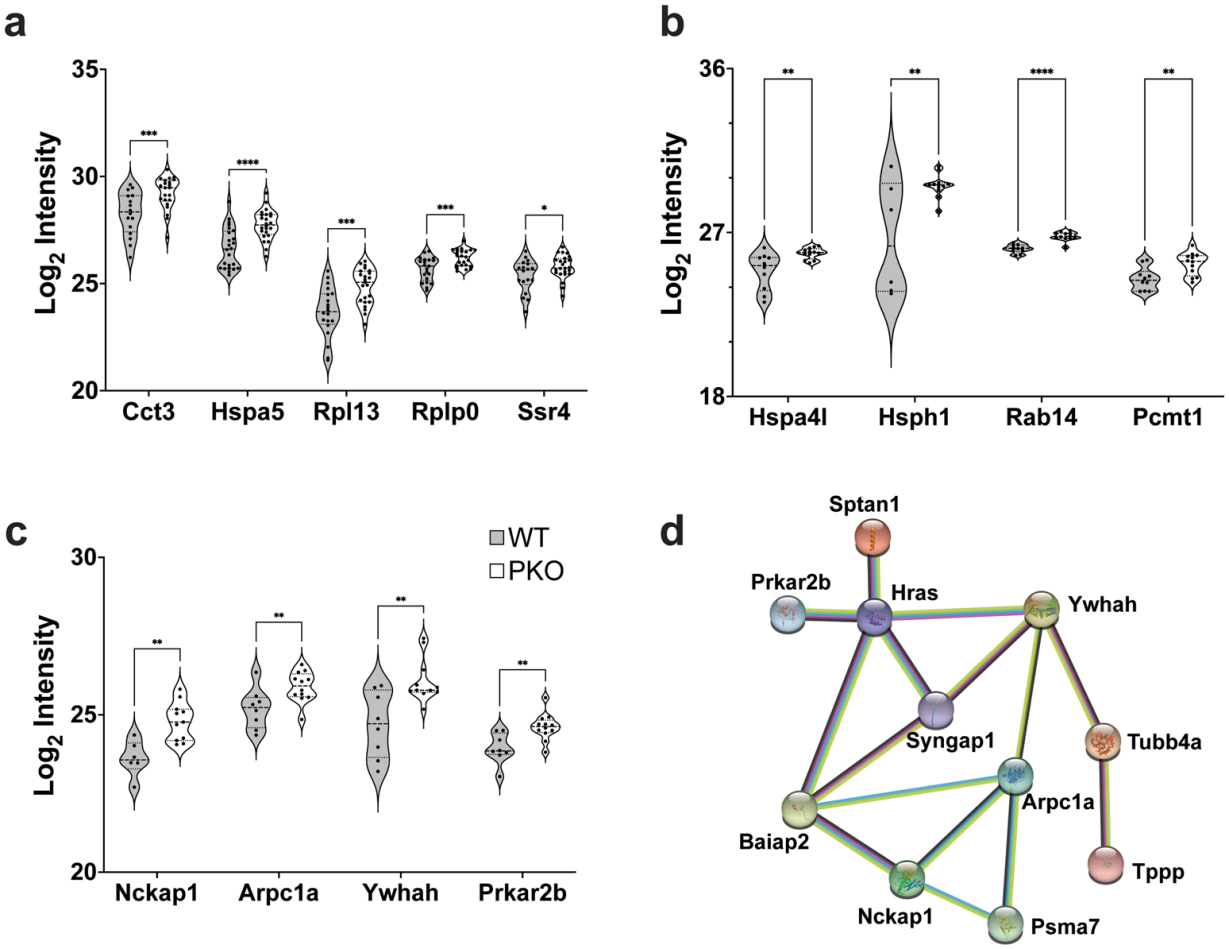
Loss of parkin affects pathways of neurodegeneration and ubiquitin-like conjugation in liver mitochondria and protein repair, aggregate mitigation, and signal transduction in striatal synaptic mitochondria. Functional annotation of DE protein lists in (**a**) hepatic or (**b**–**c**) striatal synaptic mitochondria by DAVID and expression of individual proteins in these pathways quantified in our analysis is presented as the log_2_ of intensity for each protein. Significance was determined using multiple t-tests with a Benjamini Hochberg correction (asterisks indicate the adjusted *p *value; *p < 0.05 and **p < 0.01). (**d**) The STRING database was used in network analysis of the striatal synaptic mitochondrial DE proteins. Image was created using the STRING^79^ database (version 11.4; https://string-db.org).

Functional annotation enrichment of hepatic mitochondrial DE proteins using The Database for Annotation, Visualization and Integrated Discovery (DAVID)^76–78^ revealed under parkin insufficiency, a cluster of eight proteins that are involved in ubiquitin-like conjugation processes (Fig. 3a). The complete output of the functional clustering is provided in Supplementary Table S6.

### Loss of parkin causes the upregulation of proteins involved in protein repair and aggregate mitigation as signal transduction processes in synaptic mitochondria

Similar to hepatic mitochondria, we identified proteins that were altered by loss of parkin in synaptic mitochondrial isolates. Pathway analysis and annotation revealed that several of these proteins were related to mechanisms of protein aggregate mitigation, protein repair, and signal transduction mechanisms. Among those are four proteins related to protein repair and aggregate mitigation, all of which displayed significantly increased abundance upon loss of parkin (Fig. 3b). Similarly displaying increased expression under parkin insufficiency were four proteins annotated as involved in signal transduction (Fig. 3c). The complete output of functional clustering is provided in Supplementary Table S7. Network analysis using the STRING database revealed connections between several of the proteins identified as DE in synaptic mitochondrial isolates (Fig. 3d), many of which are impacted in neurological disorders.

### Liver mitochondrial proteome dynamics in the context of parkin deficiency

**Figure 4:**
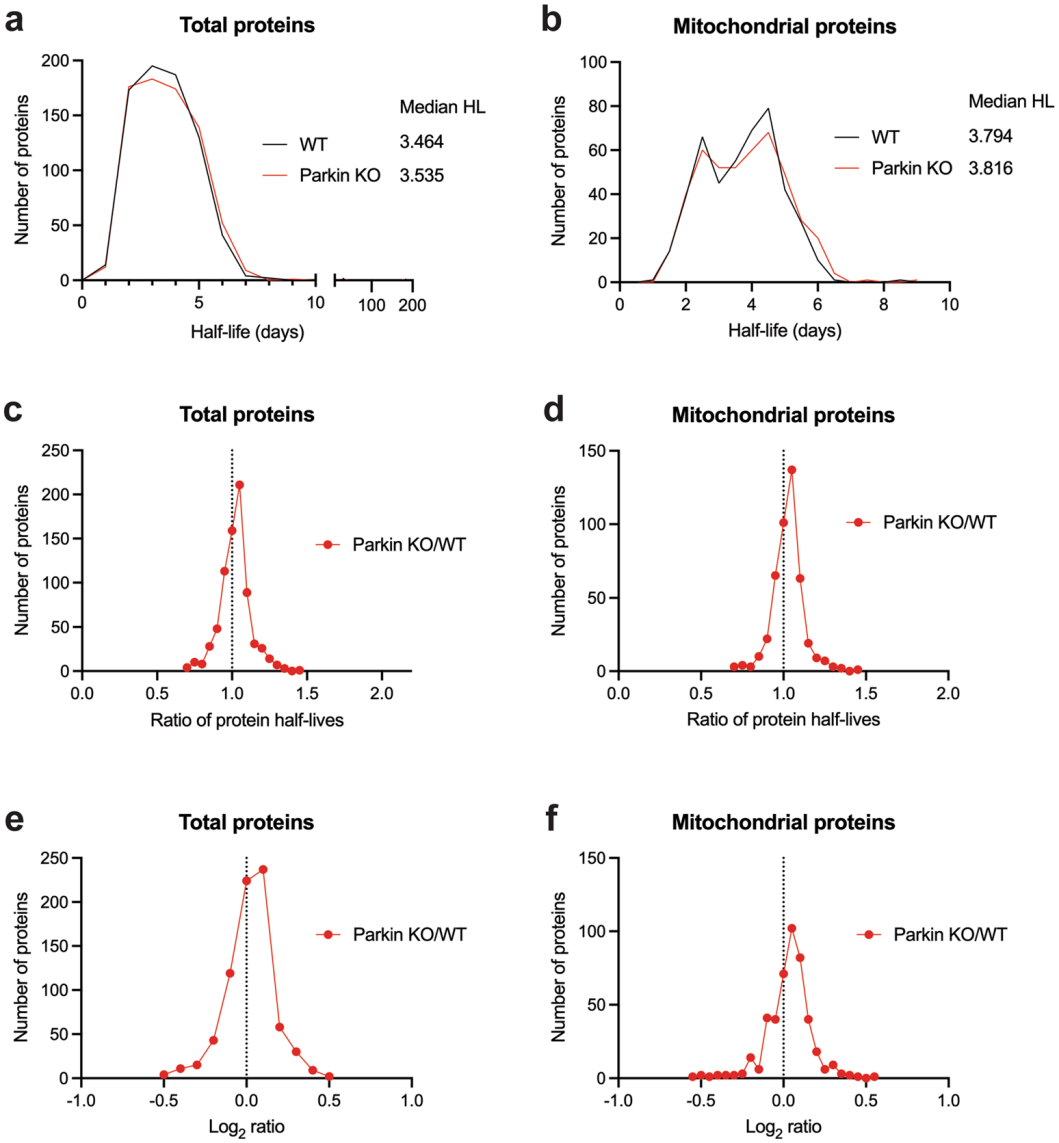
Parkin deficiency increases liver mitochondrial protein half-lives. Protein half-life distribution and median for WT (black) and PKO (red) as determined by mass spectrometry and Topograph on (**a**) total or (**b**) mitochondrial annotated proteins from liver mitochondrial preparations. Distribution of PKO/WT protein half-life ratio (**c**, **d**) or log_2_ ratio (**e**, **f**) for total (**c**, **e**) or mitochondrial annotated proteins.

To investigate the effects of parkin deficiency on liver mitochondrial protein turnover rates in vivo, we performed stable isotope metabolic labeling in mice with a synthetic diet containing ^3^H_2_-leucine. Mice were acclimatized to the synthetic diet for 21 days and then placed on a deuterated-leucine diet at 73, 78, 83, and 87 days of age. At 90 days of age, after the 17-, 12-, 7-, or 3-days stable isotope labeling, liver mitochondria were isolated, protein lysates were trypsin digested, and the resultant peptides were quantified and used for shotgun proteomics followed by protein synthesis measurements with Topograph software^72^. The proteomic analysis of turnover rates for liver mitochondria revealed a similar distribution in protein half-lives between WT and parkin KO mice (Fig. 4a, Supplementary Table S8). The median half-life of 752 proteins in the WT liver mitochondria was 3.46 days, whereas it was 3.54 days in the PKO liver mitochondria. Of these 752 proteins, 449 were annotated as true mitochondrial proteins using MitoMiner, these proteins also exhibited similar distributions in both PKO and WT animals (Fig. 4b, Supplementary Table S9). The median half-life of 449 mitochondrial proteins in the WT liver was 3.79 days, whereas it was 3.82 days in the PKO liver. Upon comparison of the ratio of half-lives of each individual protein in PKO versus WT, the mean ratio of PKO/WT is greater than 1.0 by 2.2% (total proteins, Fig. 4c) and 2.8% (mitochondrial proteins, Fig. 4d). The overall percentage of proteins exhibiting a change in half-life greater than the mean ratio were 53.1% and 50.6% of total and mitochondrial proteins, respectively, suggesting at best a small overall increase in liver mitochondrial protein turnover rates with parkin deficiency. The mean log_2_ ratio of PKO/WT is 0.025 (total proteins, Fig. 4e) and 0.033 (mitochondrial proteins, Fig. 4f). These changes indicate that the mitochondrial proteins in the liver have a slightly slower turnover (longer half-lives) in the absence of parkin. Notably, this minor increase in half-life was not accompanied by a change in total ubiquitin in the liver mitochondrial isolated from PKO mice (Supplementary Fig. 5).

### Synaptic mitochondrial proteome dynamics in the context of parkin deficiency

**Figure 5:**
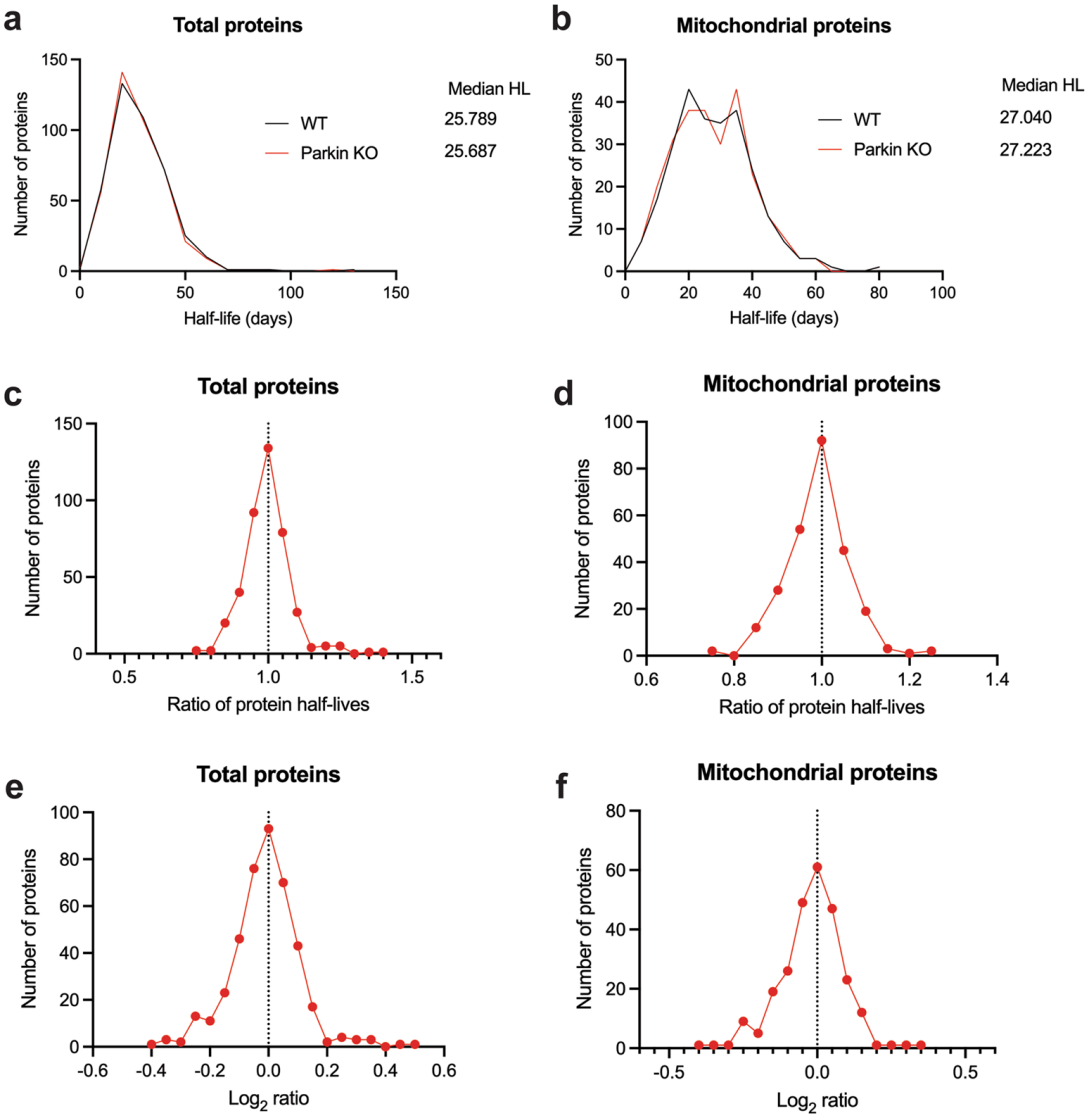
Loss of parkin produces a minimal overall effect on turnover of striatal synaptic mitochondrial proteins. Protein half-life distribution and median for WT (black) and PKO (red) as determined by mass spectrometry and Topograph on (**a**) total or (**b**) mitochondrial annotated proteins from striatal synaptic mitochondrial preparations. Distribution of PKO/WT protein half-life ratio (**c**, **d**) or log_2_ ratio (**e**, **f**) for total (**c**, **e**) or mitochondrial annotated proteins.

In addition to liver mitochondria, striatal synaptic mitochondria were also isolated from the same mice as above. The proteomics analysis of turnover rates for synaptic mitochondria revealed a similar distribution in protein half-lives between WT and PKO mice (Fig. 5a, Supplementary Table S10). The median half-life of 412 proteins in the WT synaptic mitochondria was 25.8 days, whereas it was 25.7 days in the PKO synaptic mitochondria. Of these 412 proteins, 258 were annotated as mitochondrial proteins using MitoMiner, which similar to liver displayed similar distributions in WT and PKO animals (Fig. 5b, Supplementary Table S11). The median half-life of 258 synaptic mitochondrial proteins in the WT striatum was 27.0 days, whereas it was 27.2 days in the PKO synaptic mitochondria. Upon comparison of the ratio of half-lives of each individual protein in PKO versus WT, the mean ratio of PKO/WT is approximately 1.0, only lower by 0.5% (total proteins, Fig. 5c) and 0.9% (mitochondrial proteins, Fig. 5d), suggesting no change in synaptic mitochondrial protein turnover rates with parkin deficiency. The mean log_2_ ratio of parkin KO/WT is − 0.011 (total proteins, Fig. 5e) and -0.017 (mitochondrial proteins, Fig. 5f). These changes indicate that the mitochondrial proteins have a similar turnover (similar half-lives) in the absence of parkin.

### Pathway analysis of the effect of parkin deficiency on liver mitochondrial proteome dynamics

**Figure 6:**
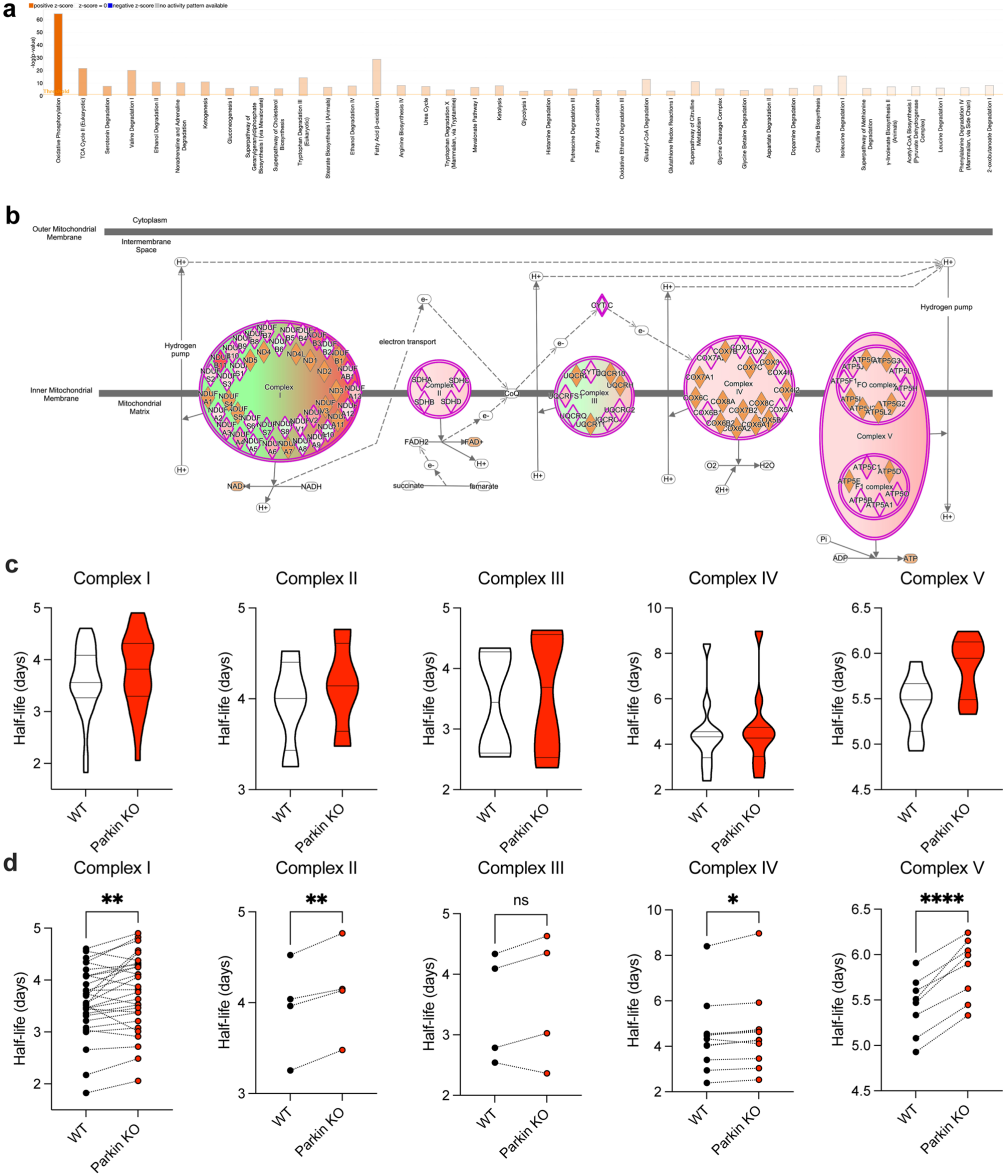
Parkin insufficiency has pronounced effect on respiratory chain components. (**a**) IPA “Canonical Pathways” identified to exhibit significant activation scores. Magnitude of activation z-score is represented by color saturation gradient (i.e., the greater the magnitude the darker the color). (**b**) Details of the “Oxidative Phosphorylation” pathway analysis predicts positive activation scores for many respiratory complex subunits. Image modified from IPA output. (**c**) Violin plots illustrating overall average half-life of respiratory chain complexes in liver mitochondria. (**d**) Trajectory plots of individual protein subunits included in the averages for whole complexes in (**c**). Statistical significance was determined by two-tailed paired t-test (*p < 0.05, **p < 0.01, ****p < 0.0001).

To identify protein pathways that are differentially regulated by parkin deficiency, we performed canonical pathway analysis of our dataset using IPA. The mitochondrial proteins sorted to 30 canonical metabolic pathways exhibiting an absolute value z-score greater than 1 and a − log(*p* value) greater than 1.3 (i.e. *p* < 0.05; Fig. 6a, Supplementary Table S12). The top pathway according to activation z-score was oxidative phosphorylation exhibiting positive activation (z-score = 5.0) based on the increased half-lives of the subunits of the electron transport chain (ETC) complexes I–V in the absence of parkin (Fig. 6b). Except for complex IV, the median half-lives of the subunits for each of the ETC complexes were higher in liver mitochondria from PKO as compared to WT mice (complex V, + 0.46 > complex I, + 0.26 > complex III, + 0.25 > complex II, + 0.14 > complex IV, -0.05), suggesting reduced turnover (Fig. 6c). Further supporting this finding, the individual half-lives for the majority of the subunits for each of the ETC complexes were elevated in PKO mice (Fig. 6d).

### Network analysis of the shortest- and longest-lived liver mitochondrial proteins in parkin KO mice

**Figure 7:**
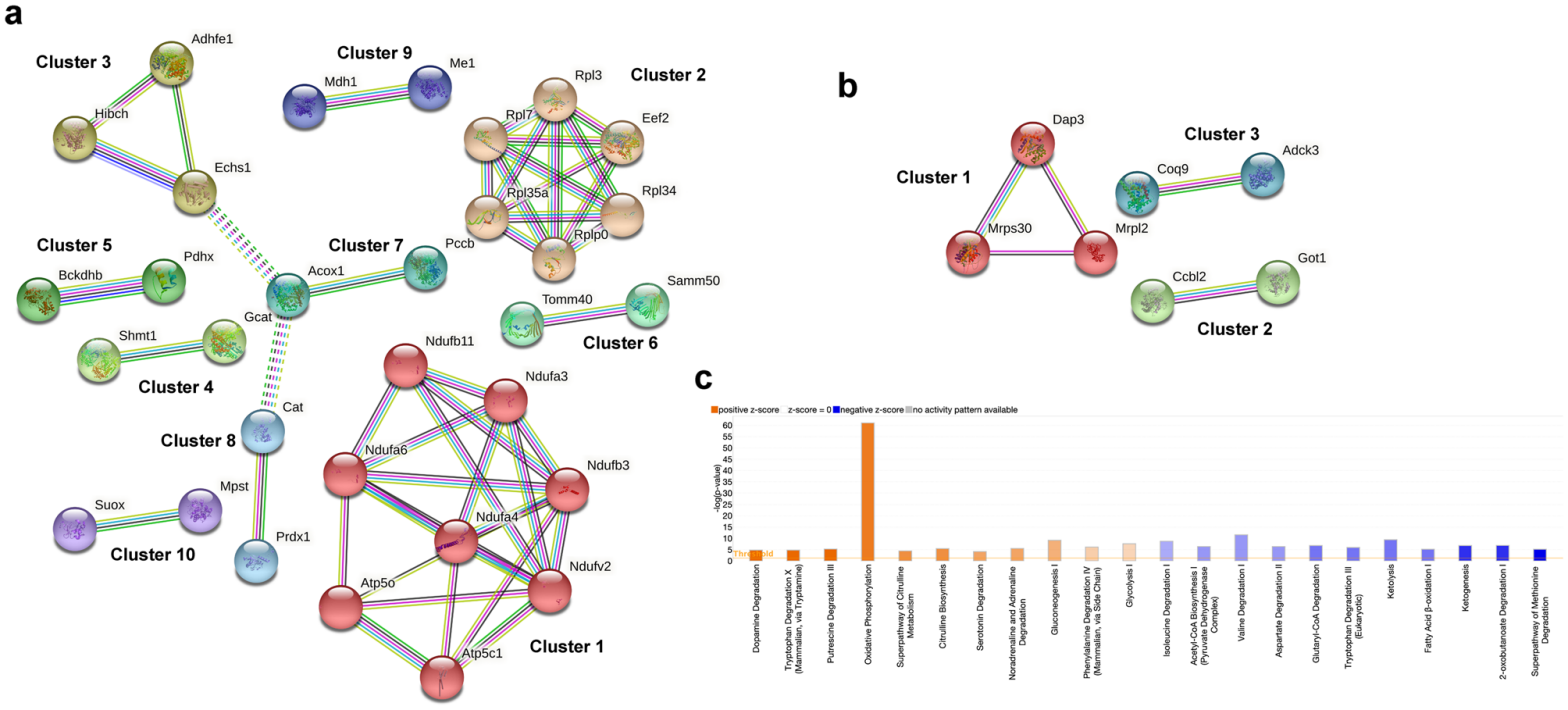
In striatal synaptic mitochondria loss of parkin alters pathways related to bioenergetics, protein translation, and neurotransmitter metabolism. Network analysis of mitochondrial annotated proteins from striatal synaptic mitochondrial preparation using the STRING database that were determined to display elevated (**a**) or decreased (**b**) half-life. Clusters were determined using Markov clustering. Images in (**a**–**b**) were created using the STRING^79^ database (version 11.4; https://string-db.org) (**c**) IPA “Canonical Pathways” that exhibited significant activation scores. Magnitude of activation z-score is represented by color saturation gradient (i.e., the greater the magnitude the darker the color), while directionality is represented by color (i.e., orange = positive and blue = negative). Image modified from IPA output.

The mitochondrial proteins exhibiting half-lives that increased (70 proteins, slower turnover) or decreased (31 proteins, faster turnover) by at least 10% in PKO mice as compared to WT mice were analyzed using the search tool for retrieval of interacting genes (STRING) to acquire protein–protein interaction (PPI) networks. Figure 7a shows the 10 significant clusters that were found in the slower turnover PPI network analysis using STRING MCL (Markov) clustering. Functional annotation of the top PPI network cluster (Fig. 7a, cluster 1) revealed enrichment of three Reactome Pathways, complex I biogenesis (MMU-6799198), respiratory electron transport (MMU-611105), and respiratory electron transport, ATP synthesis by chemiosmotic coupling, and heat production by uncoupling proteins (MMU-163200). Figure 7b shows the 3 significant clusters that were found in the faster turnover PPI network analysis using STRING MCL clustering. Functional annotation of the top PPI network cluster (Fig. 7b, cluster 1) revealed enrichment of two Reactome Pathways, mitochondrial translation elongation (MMU-5389840) and mitochondrial translation termination (MMU-5419276). Of note, while the proteins showing faster turnover are involved in mitochondrial translation, cluster 2 in Fig. 7a highlights that several ribosomal proteins showed slower turnover.

### Pathway and STRING network analysis of the effect of parkin deficiency on synaptic mitochondrial proteome dynamics

Although overall there was no global effect of parkin deficiency, there were pathways that are differentially regulated by parkin deficiency that were revealed by IPA. The mitochondrial proteins sorted to 18 canonical metabolic pathways exhibiting an absolute value z-score greater than 1 and a − log(*p*-value) greater than 1.3 (i.e. *p* < 0.05; Fig. 7c, Supplementary Table S13). The top pathway according to activation z-score was “Dopamine Degradation” exhibiting positive activation (z-score = 2.0), quite interesting as the striatum is dopamine rich.

### Comparison between liver and synaptic mitochondria

**Figure 8:**
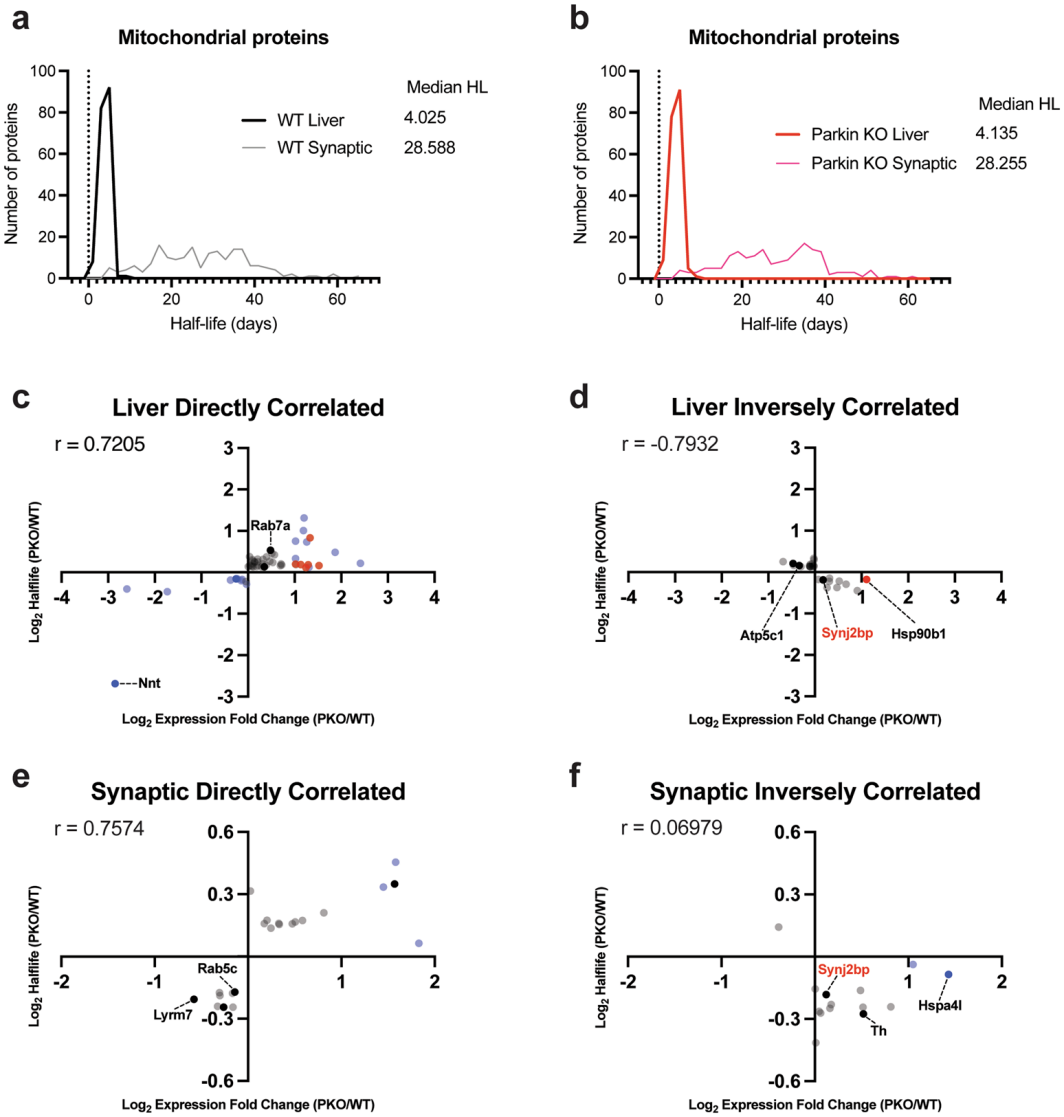
Striatal synaptic mitochondria proteins are longer lived but less susceptible to loss of parkin than those of liver mitochondria. Distribution of mitochondrial protein half-life between tissues for (**a**) WT and (**b**) PKO demonstrating, independent of genotype, striatal synaptic mitochondrial half-life is longer than liver mitochondria. Correlation of protein abundance and half-life for (**c**, **d**) liver mitochondria and (**e**, **f**) striatal synaptic mitochondria. Proteins were segregated into those exhibiting (**c**, **e**) direct or (**d**, **f**) inverse correlation. (**c**–**f**) Dots represent proteins that were derived from the list of DE proteins (abundance, blue), differential half-life (turnover, black dots), or shared (both, red). Gene symbols in red represent proteins that overlap in liver and striatal synaptic lists.

Of the 449 (liver) and 258 (synaptic) proteins annotated as mitochondrial proteins using MitoMiner, 184 mitochondrial proteins were found in both datasets (Supplementary Table S14). The proteomic analysis of turnover rates for these 184 proteins revealed divergent distributions in protein half-lives between mitochondria isolated from the liver and synaptic mitochondria isolated from the striatum of WT mice (Fig. 8a, Supplementary Table S14). The median half-lives of these 184 proteins in the WT liver mitochondria were 4.03 days, whereas they were 28.6 days in the WT striatal synaptic mitochondria. Similarly, the median half-lives of the 184 proteins in the PKO liver mitochondria were 4.14 days, whereas they were 28.3 days in the PKO striatal synaptic mitochondria (Fig. 8b, Supplementary Table S14). Thus, the mitochondrial proteins localized in the synapse have slower turnover (longer half-lives) than when found in the liver.

### Proteins with altered half-lives display altered expression in hepatic and synaptic mitochondria

Protein abundance is a balance between protein production and destruction, and alterations in half-life can affect abundance. Hepatic or synaptic mitochondrial proteins determined to be DE in abundance or with significantly altered half-lives were used in correlation analysis to determine if the change in half-life was coincident with altered expression in PKO relative to WT. These lists were comprised of 83 and 36 proteins for hepatic and synaptic mitochondria, respectively. We first analyzed the correlations for these proteins in each tissue, revealing Pearson’s correlation coefficients (r) of 0.5907 for hepatic, and 0.5178 for synaptic. We then partitioned these lists into proteins that were directly or inversely correlated, improving the overall correlation (Fig. 8c–f, Supplementary Table S15). Interestingly, in both hepatic and synaptic mitochondria approximately two-thirds of the proteins with altered abundance or half-life were directly correlated with the remaining one-third inversely correlated. Directly correlated proteins in liver include Nnt reduced expression and shorter half-life in PKO (implying increased destruction relative to production) and Rab7a with increased expression and half-life in PKO (implying decreased destruction relative to production). Nnt facilitates the production of NADPH from NADH and NADP, and is associated with regulating redox state^80^. Notably, Rab7a and parkin exhibit reciprocal regulation^81,82^ and loss of parkin in vitro is associated with elevated Rab7 expression^82^. From synaptic mitochondria Lyrm7, a Complex III assembly factor which when mutated causes mitochondrial Complex III deficiency^83^, exhibited decreased expression and shorter half-lives. Among inversely correlated hepatic mitochondrial proteins were Atp5c1, with reduced expression and increased half-life, and Hsp90b1 with increased expression and decreased half-life. Atp5c1 encodes the gamma subunit of the catalytic core of Complex V^84^, and mutations are associated with a form of late onset AD^85^. Hsp90b1 is interesting because Hsp90 family proteins are molecular chaperones that aid in protein folding and mitigating alpha-synuclein pathology^86,87^. In synaptic mitochondria tyrosine hydroxylase, which catalyzes the rate limiting step of dopamine production, and Hspa4l, a protein that mitigates alpha-synuclein aggregation^88^, are notable for increased abundance while their half-lives are decreased. Only one protein, synaptojanin 2-binding protein (Synj2bp) exhibited inverse correlation in both. Interestingly, expression of Synj2b (also known as Omp25), which functions in recruiting cell adhesions molecules to mitochondria^89^, was elevated in both mitochondrial populations.

## Discussion

The results of our study demonstrate in vivo differences in protein abundance and turnover rates within the mitochondrial proteomes derived from liver or striatal synapses, as well as tissue-specific effects of loss of parkin, offering insight into potential novel downstream targets regulated by parkin activity. Specifically, we report that loss of parkin leads to an overall general increase in protein abundance in both liver and striatal synaptic mitochondria, specifically significant differences in expression of 38 and 12 proteins, respectively. In line with this, loss of parkin in either tissue resulted in a subtle increase in overall protein half-life that was more readily apparent in hepatic mitochondrial than striatal synaptic. Importantly, overall protein half-lives were longer in striatal synaptic mitochondria (median half-life ~ 25.7 days) than of hepatic mitochondria (median half-life ~ 3.5 days). We did not find a generalized effect of parkin deficiency on protein abundance or half-life, pointing to a largely negligible role of parkin in mitochondria homeostasis, at least in the liver and striatal synaptosomes. Although it is tempting to compare protein abundance changes between hepatic and striatal synaptic mitochondria our study is limited by acquisition of raw data from mitochondria derived from each tissue using different instrumentation.

Typically associated with mitochondrial damage response, the E3-ubiquitin ligase parkin is an effector of mitophagy and when mutated results in autosomal recessive juvenile PD. Historically most studies investigate parkin function and loss thereof in the context of mitophagy, which is driven in experimental systems through dramatic loss of mitochondrial membrane potential such as treatment of cells with carbonyl cyanide m-chlorophenyl hydrazone (CCCP). While an essential mitigator when mitochondrial damage occurs, mitophagy is also critical for homeostatic (i.e*.* basal) mitochondrial turnover aiding in the regulation of many cellular and physiological processes including cellular metabolism, calcium buffering, regulation of REDOX state, clearance of mitochondria during red blood cell maturation, neurotransmitter release, and cell survival^90^. Congruent with the importance of parkin and mitophagy in PD, we determined that parkin insufficiency resulted in changes that are associated with pathways of neurodegeneration. However, whereas mitophagy is thought to affect the mitochondrion as a whole, we only found effects on certain proteins. Specifically, loss of parkin in our study correlated with alterations in the abundance of synaptic mitochondrial proteins related to protein aggregate clearance and repair mechanisms including Hsph1*,* Hspa4l^88^*,* and Pcmt1^91^*.* Importantly, these have been shown to act on alpha-synuclein^88^, the major proteopathic component of Lewy Bodies which contributes to the neuropathological damage in PD^37,92^. Pcmt1 additionally functions in regulating mitochondrial morphology which is disrupted in PD^91^. Other proteins involved in myelination and cytoskeleton stability also appeared to be upregulated, perhaps as a response to myelin damage and/or improper vesicle trafficking which is seen in certain neurodegenerative diseases including PD^34,93^. In the hepatic mitochondrial proteome, alterations in the abundance of proteins related to pathways of neurodegeneration were observed. A majority among these were multiple subunit components of respiratory Complex I, which is theorized to be to particularly susceptible to proteopathic damage^92,94,95^ and is the target of 1-methyl-4-phenyl-1,2,3,6-tetrahydropyridine, MPTP, a neurotoxin which results in a PD-like syndrome in people^96^, and used to model PD in rodents, and its analogues^43,97–99^.

To date several studies have explored the effects of parkin insufficiency on basal mitophagy in vitro (reviewed in^100^), yet few, limited to *Drosophila* models, have investigated its importance under homeostatic conditions in vivo*,* the most prominent of which report conflicting findings^23,53^. A landmark study by Vincow et al.^23^, employing a metabolic labeling proteomics approach comparable to the one we used herein, reported both significant increases in mitochondrial protein half-lives from parkin loss of function mutant flies and selective turnover of respiratory chain components. In contrast, a more recent study suggested although basal mitophagy is plentiful in *Drosophila* in either larval epidermis or CNS but not adult flight wing muscle, it is only minimally impacted by loss of parkin function^53^. Notably, a key difference between these studies is the method of measuring mitophagy. Methods aside, a possible explanation for this discrepancy may be selective vulnerability in certain types and subpopulations of cells^101,102^ as reported by Cackovic et al.^32^. In support of a model in which parkin serves a minor role in basal mitophagy we observed only minor elevations in the median half-lives of mitochondrial proteomes of both liver mitochondria and striatal synaptic mitochondria. In line with this, basal mitophagy was shown to occur despite the absence of parkin activation in tissues of high energy consumption including brains of PINK1 KO mice^103^, which may be the result of an alternate pathway regulated by Mul1^28,29^. Furthermore additional PINK1/parkin independent pathways that may function under homeostatic conditions have also been described^104,105^. The presence of these alternate pathways has been theorized to contribute to the poor reflection of rodent PD models in the neuropathological phenotypes observed in patients bearing loss of function mutations in either PINK1 or parkin^48,106^. Although we did not directly investigate this, we did observe elevated abundance of proteins involved in ubiquitin-like conjugation processes in hepatic mitochondrial proteomes, yet further investigation would be required to determine mechanistically if alternative pathways are acting and to what degree in this system. Together with only modest changes observed in protein abundance and turnover rates under parkin insufficiency our study lends support to recent reports suggesting that basal mitophagy is largely a parkin independent process^53,103,104,107^. With our intentional focus on mitochondrial isolates, our study does not comprehensively explore the extra-mitochondrial functions of parkin, which could be the subject of future investigations. Furthermore, although the use of tri-deuterated leucine is an established method of metabolic labeling, a limitation of this is the presence of peptides in the analysis that do not contain leucine and thus while they contribute to the expression analysis do not contribute to half-life determination.

One particularly interesting aspect of our study is the observation of tissue-specific protein turnover timescales between liver and striatal synaptic mitochondria, such that synaptic mitochondria exhibited a greater lifespan. A likely explanation for this difference is the need for mitochondria to travel long distances from the soma to the synaptic terminals where they function^108,109^. Importantly, this may also in part explain why synaptic mitochondria are particularly susceptible to damage which in turn leads to synaptic failure described is neurodegeneration^109^. Our findings are supported by earlier studies in *Drosophila* reporting differential mitochondrial turnover rates in different fly tissues^52^ and in distinct cell types during autophagic flux in *C. elegans*^54^. This suggests that tissue specific regulation of basal mitophagy which may result from differential expression of upstream regulators. Our observation of altered sirtuin signaling predicted in hepatic mitochondrial samples by IPA supports this. Sirtuin signaling is central regulator of mitochondrial homeostasis contributing to mitophagy, fission fusion dynamics, and biogenesis via several pathways^110,111^. Our results may also suggest a feedback mechanism since sirtuin signaling displayed a negative activation score under conditions of parkin insufficiency, however, this was not directly addressed. In addition to sirtuin signaling, other potential regulators may also influence the differences in tissue specific mitochondrial turnover rates. One notable candidate of recent interest is the membrane associated ring-CH-type finger 5 (Marchf5, also known as MITOL, a mitochondrial resident E3 ubiquitin ligase)^21^ which when depleted results in altered mitochondrial structure and promotes oxidative stress^112^. Canonical mitophagy relies on a positive feedback loop between parkin and PINK1, and MITOL was recently implicated as a potential initiation seed for recruiting parkin in a PINK1 dependent manner^21^. Although detected in our proteomics, our study design did not afford direct comparisons between tissue types under normal or parkin deficient conditions to assess if differential expression of MITOL is between hepatic and striatal synaptic mitochondria may contribute to tissue specific turnover rates. Further studies combining parkin insufficiency and mitochondrial stress will be of particular interest to further characterize tissue specific roles of parkin.

In summary our study revealed both differential basal turnover rates of hepatic and synaptic mitochondrial proteins as well as differential effects of loss of parkin. Our findings suggest that parkin function makes only modest contributions to basal mitophagy in vivo, and this is more pronounced in liver than in striatal synapses. Specifically, we report the median half-life of the synaptic mitochondrial proteome is substantially longer than that of hepatic mitochondria, a finding that supports the importance of synaptic mitochondria in neurodegenerative disorders. Notably, loss of parkin was associated with general increased protein abundance that correlated with reduced turnover (increased protein half-life). However, these changes were minor and support a recent paradigm shift in the field of mitophagy lending support to a model in which parkin-dependent mitophagy plays a modest role in basal mitophagy, suggesting additional studies should focus efforts on studying the effects of parkin insufficiency in the context of mitochondrial stress or damage. Finally, our study identified potential novel targets of parkin activity, opening new avenues for continued investigation into mechanisms of action for parkin.

## Supplementary Information

Supplementary Figure 1.

Supplementary Figure 2.

Supplementary Figure 3.

Supplementary Figure 4.

Supplementary Figure 5.

Supplementary Tables.

## Data Availability

The mass spectrometry proteomics data presented herein have been deposited to the ProteomeXchange Consortium via the PRIDE^113^ partner repository with the dataset identifier PXD037940 (abundance) and PXD037993 (turnover).

## References

[1] Pathak D (2015). The role of mitochondrially derived ATP in synaptic vesicle recycling. J. Biol. Chem..

[2] Harris J, Jolivet R, Attwell D (2012). Synaptic energy use and supply. Neuron.

[3] Südhof TC (2012). Calcium control of neurotransmitter release. Cold Spring Harb. Perspect. Biol..

[4] Datta S, Jaiswal M (2021). Mitochondrial calcium at the synapse. Mitochondrion.

[5] Chen G, Mitophagy GKOK (2020). An emerging role in aging and age-associated diseases. Front. Cell Dev. Biol..

[6] Norat P (2020). Mitochondrial dysfunction in neurological disorders: Exploring mitochondrial transplantation. npj Regen. Med..

[7] Seager R, Lee L, Henley JM, Wilkinson KA (2020). Mechanisms and roles of mitochondrial localisation and dynamics in neuronal function. Neuronal Signal..

[8] Youle RJ, van der Bliek AM (2012). Mitochondrial fission, fusion, and stress. Science.

[9] Eckl E-M, Ziegemann O, Krumwiede L, Fessler E, Jae LT (2021). Sensing, signaling and surviving mitochondrial stress. Cell. Mol. Life Sci..

[10] Bolstad BM, Irizarry RA, Åstrand M, Speed TP (2003). A comparison of normalization methods for high density oligonucleotide array data based on variance and bias. Bioinformatics.

[11] Johri A, Beal MF (2012). Mitochondrial dysfunction in neurodegenerative diseases. J. Pharmacol. Exp. Ther..

[12] Liu M (2019). Inhibition of calpain protects against tauopathy in transgenic P301S tau mice. J. Alzheimer’s Dis..

[13] Zündorf G, Reiser G (2011). Calcium dysregulation and homeostasis of neural calcium in the molecular mechanisms of neurodegenerative diseases provide multiple targets for neuroprotection. Antioxid. Redox Signal..

[14] Adav SS, Park JE, Sze SK (2019). Quantitative profiling brain proteomes revealed mitochondrial dysfunction in Alzheimer’s disease. Mol. Brain.

[15] Pathak D, Berthet A, Nakamura K (2013). Energy failure: Does it contribute to neurodegeneration?. Ann. Neurol..

[16] Parihar MS, Brewer GJ (2007). Mitoenergetic failure in Alzheimer disease. Am. J. Physiol.-Cell Physiol..

[17] Moreno-Sánchez R (2013). Reactive oxygen species are generated by the respiratory complex II - Evidence for lack of contribution of the reverse electron flow in complex I. FEBS J..

[18] Ding WX, Yin XM (2012). Mitophagy: Mechanisms, pathophysiological roles, and analysis. Biol. Chem..

[19] Koyano F, Matsuda N (2015). Molecular mechanisms underlying PINK1 and Parkin catalyzed ubiquitylation of substrates on damaged mitochondria. Biochim. Biophys. Acta Mol. Cell Res..

[20] Koyano F (2014). Ubiquitin is phosphorylated by PINK1 to activate parkin. Nature.

[21] Koyano F, Yamano K, Kosako H, Tanaka K, Matsuda N (2019). Parkin recruitment to impaired mitochondria for nonselective ubiquitylation is facilitated by MITOL. J. Biol. Chem..

[22] Iguchi M (2013). Parkin-catalyzed ubiquitin-ester transfer is triggered by PINK1-dependent phosphorylation. J. Biol. Chem..

[23] Vincow ES (2013). The PINK1-Parkin pathway promotes both mitophagy and selective respiratory chain turnover in vivo. Proc. Natl. Acad. Sci. U. S. A..

[24] Kazlauskaite A (2015). Binding to serine 65-phosphorylated ubiquitin primes Parkin for optimal PINK 1-dependent phosphorylation and activation. EMBO Rep..

[25] Youle RJ, Narendra DP (2011). Mechanisms of mitophagy. Nat. Rev. Mol. Cell Biol..

[26] Bingol B, Sheng M (2016). Mechanisms of mitophagy: PINK1, Parkin, USP30 and beyond. Free Radic. Biol. Med..

[27] Shen JL, Fortier TM, Wang R, Baehrecke EH (2021). Vps13D functions in a Pink1-dependent and Parkin-independent mitophagy pathway. J. Cell Biol..

[28] Yun J (2014). MUL1 acts in parallel to the PINK1/parkin pathway in regulating mitofusin and compensates for loss of PINK1/parkin. Elife.

[29] Rojansky R, Cha MY, Chan DC (2016). Elimination of paternal mitochondria in mouse embryos occurs through autophagic degradation dependent on PARKIN and MUL1. Elife.

[30] Li J (2015). Mitochondrial outer-membrane E3 ligase MUL1 ubiquitinates ULK1 and regulates selenite-induced mitophagy. Autophagy.

[31] Cummins N, Tweedie A, Zuryn S, Bertran-Gonzalez J, Götz J (2019). Disease-associated tau impairs mitophagy by inhibiting Parkin translocation to mitochondria. EMBO J..

[32] Cackovic J (2018). Vulnerable parkin loss-of-function drosophila dopaminergic neurons have advanced mitochondrial aging, mitochondrial network loss and transiently reduced autophagosome recruitment. Front. Front. Cell. Neurosci..

[33] Karbowski M, Neutzner A (2012). Neurodegeneration as a consequence of failed mitochondrial maintenance. Acta Neuropathol..

[34] Sheehan P, Yue Z (2019). Deregulation of autophagy and vesicle trafficking in Parkinson’s disease. Neurosci. Lett..

[35] Madsen DA, Schmidt SI, Blaabjerg M, Meyer M (2021). Interaction between Parkin and α-Synuclein in PARK2-mediated Parkinson’s disease. Cells.

[36] Wilson RS, Leurgans SE, Boyle PA, Schneider JA, Bennett DA (2010). Neurodegenerative basis of age-related cognitive decline (e–Pub ahead of print)(CME). Neurology.

[37] Reish HEA, Standaert DG (2015). Role of α-synuclein in inducing innate and adaptive immunity in Parkinson disease. J. Parkinsons. Dis..

[38] Lu L (2006). Regional vulnerability of mesencephalic dopaminergic neurons prone to degenerate in Parkinson’s disease: A post-mortem study in human control subjects. Neurobiol. Dis..

[39] Minakaki G, Krainc D, Burbulla LF (2020). The convergence of alpha-synuclein, mitochondrial, and lysosomal pathways in vulnerability of midbrain dopaminergic neurons in Parkinson’s disease. Front. Cell Dev. Biol..

[40] Kamath T (2022). Single-cell genomic profiling of human dopamine neurons identifies a population that selectively degenerates in Parkinson’s disease. Nat. Neurosci..

[41] Fu H, Hardy J, Duff KE (2018). Selective vulnerability in neurodegenerative diseases. Nat. Neurosci..

[42] Schneider JS, Yuwiler A, Markham CH (1987). Selective loss of subpopulations of ventral mesencephalic dopaminergic neurons in the monkey following exposure to MPTP. Brain Res..

[43] Varastet M, Riche D, Maziere M, Hantraye P (1994). Chronic MPTP treatment reproduces in baboons the differential vulnerability of mesencephalic dopaminergic neurons observed in Parkinson’s disease. Neuroscience.

[44] Yamada T, McGeer PL, Baimbridge KG, McGeer EG (1990). Relative sparing in Parkinson’s disease of substantia nigra dopamine neurons containing calbindin-D28K. Brain Res..

[45] Klein C, Westenberger A (2012). Genetics of Parkinson’s disease. Cold Spring Harb. Perspect. Med..

[46] Wallings RL, Tansey MG (2019). LRRK2 regulation of immune-pathways and inflammatory disease. Biochem. Soc. Trans..

[47] Liu J, Zhang C, Hu W, Feng Z (2018). Parkinson’s disease-associated protein Parkin: An unusual player in cancer. Cancer Commun..

[48] Konnova EA, Swanberg M (2018). Animal models of Parkinson’s disease. Park. Dis. Pathog. Clin. Asp..

[49] Almikhlafi MA (2020). Deletion of DJ-1 in rats affects protein abundance and mitochondrial function at the synapse. Sci. Rep..

[50] Palacino JJ (2004). Mitochondrial dysfunction and oxidative damage in parkin-deficient mice. J. Biol. Chem..

[51] Gispert S (2009). Parkinson phenotype in aged PINK1-deficient mice is accompanied by progressive mitochondrial dysfunction in absence of neurodegeneration. PLoS ONE.

[52] Kim YY (2019). Assessment of mitophagy in mt-Keima drosophila revealed an essential role of the PINK1-Parkin pathway in mitophagy induction in vivo. FASEB J..

[53] Lee JJ (2018). Basal mitophagy is widespread in Drosophila but minimally affected by loss of Pink1 or parkin. J. Cell Biol..

[54] Chapin HC, Okada M, Merz AJ, Miller DL (2015). Tissue-specific autophagy responses to aging and stress in C. elegans. Aging (Albany NY).

[55] Goldberg MS (2003). Parkin-deficient mice exhibit nigrostriatal deficits but not loss of dopaminergic neurons. J. Biol. Chem..

[56] Graham JM (1999). Purification of a crude mitochondrial fraction by density-gradient centrifugation. Curr. Protoc. Cell Biol..

[57] Stauch KL, Purnell PR, Fox HS (2014). Quantitative proteomics of synaptic and nonsynaptic mitochondria: Insights for synaptic mitochondrial vulnerability. J. Proteome Res..

[58] Wiśniewski JR (2017). Filter-aided sample preparation: The versatile and efficient method for proteomic analysis. Methods in Enzymology.

[59] Scopes RK (1974). Measurement of protein by spectrophotometry at 205 nm. Anal. Biochem..

[60] Cox J, Mann M (2008). MaxQuant enables high peptide identification rates, individualized p.p.b.-range mass accuracies and proteome-wide protein quantification. Nat. Biotechnol..

[61] Tyanova S, Temu T, Cox J (2016). The MaxQuant computational platform for mass spectrometry-based shotgun proteomics. Nat. Protoc..

[62] Shah AD, Goode RJA, Huang C, Powell DR, Schittenhelm RB (2019). Lfq-Analyst: An easy-to-use interactive web platform to analyze and visualize label-free proteomics data preprocessed with maxquant. J. Proteome Res..

[63] R: The R Project for Statistical Computing. https://www.r-project.org/.

[64] Tyanova S (2016). The Perseus computational platform for comprehensive analysis of (prote)omics data. Nat. Methods.

[65] Hoopmann MR, Finney GL, MacCoss MJ (2007). High speed data reduction, feature detection, and MS/MS spectrum quality assessment of shotgun proteomics datasets using high resolution mass spectrometry. Anal. Chem..

[66] Hoopmann MR, Maccoss MJ, Moritz RL (2012). Identification of peptide features in precursor spectra using Hardklör and Krönik. Curr. Protoc. Bioinform..

[67] Hsieh EJ, Hoopmann MR, MacLean B, MacCoss MJ (2010). Comparison of database search strategies for high precursor mass accuracy MS/MS data. J. Proteome Res..

[68] Eng JK, Jahan TA, Hoopmann MR (2013). Comet: An open-source MS/MS sequence database search tool. Proteomics.

[69] Käll L, Canterbury JD, Weston J, Noble WS, MacCoss MJ (2007). Semi-supervised learning for peptide identification from shotgun proteomics datasets. Nat. Methods.

[70] Park CY, Klammer AA, Käli L, MacCoss MJ, Noble WS (2008). Rapid and accurate peptide identification from tandem mass spectra. J. Proteome Res..

[71] Pino LK (2020). The skyline ecosystem: Informatics for quantitative mass spectrometry proteomics. Mass Spectrom. Rev..

[72] Hsieh EJ (2012). Topograph, a software platform for precursor enrichment corrected global protein turnover measurements. Mol. Cell. Proteomics.

[73] Krämer A, Green J, Pollard J, Tugendreich S (2014). Causal analysis approaches in ingenuity pathway analysis. Bioinformatics.

[74] Tissue expression of PRKN - Summary - The human protein atlas. https://www.proteinatlas.org/ENSG00000185345-PRKN/tissue.

[75] Uhlén M (2015). Tissue-based map of the human proteome. Science.

[76] Huang DW, Sherman BT, Lempicki RA (2009). Systematic and integrative analysis of large gene lists using DAVID bioinformatics resources. Nat. Protoc..

[77] Huang DW, Sherman BT, Lempicki RA (2009). Bioinformatics enrichment tools: Paths toward the comprehensive functional analysis of large gene lists. Nucleic Acids Res..

[78] Sherman BT (2022). DAVID: a web server for functional enrichment analysis and functional annotation of gene lists (2021 update). Nucleic Acids Res..

[79] Szklarczyk D (2011). The STRING database in 2011: Functional interaction networks of proteins, globally integrated and scored. Nucleic Acids Res..

[80] Lopert P, Patel M (2014). Nicotinamide nucleotide transhydrogenase (Nnt) links the substrate requirement in brain mitochondria for hydrogen peroxide removal to the thioredoxin/peroxiredoxin (Trx/Prx) system. J. Biol. Chem..

[81] Heo JM (2018). RAB7A phosphorylation by TBK1 promotes mitophagy via the PINK-PARKIN pathway. Sci. Adv..

[82] Song P, Trajkovic K, Tsunemi T, Krainc D (2016). Parkin modulates endosomal organization and function of the endo-lysosomal pathway. J. Neurosci..

[83] Fernández-Vizarra E, Zeviani M (2015). Nuclear gene mutations as the cause of mitochondrial complex III deficiency. Front. Genet..

[84] Jabs EW (1994). Chromosomal localization of genes required for the terminal steps of oxidative metabolism: Alpha and gamma subunits of ATP synthase and the phosphate carrier. Hum. Genet..

[85] Sims R (2017). Rare coding variants in PLCG2, ABI3, and TREM2 implicate microglial-mediated innate immunity in Alzheimer’s disease. Nat. Genet..

[86] Falsone SF, Kungl AJ, Rek A, Cappai R, Zangger K (2009). The molecular chaperone Hsp90 modulates intermediate steps of amyloid assembly of the Parkinson-related protein alpha-synuclein. J. Biol. Chem..

[87] Daturpalli S, Waudby CA, Meehan S, Jackson SE (2013). Hsp90 inhibits α-synuclein aggregation by interacting with soluble oligomers. J. Mol. Biol..

[88] Taguchi YV (2019). Hsp110 mitigates α-synuclein pathology in vivo. Proc. Natl. Acad. Sci. U. S. A..

[89] Hartmann C (2020). The mitochondrial outer membrane protein SYNJ2BP interacts with the cell adhesion molecule TMIGD1 and can recruit it to mitochondria. BMC Mol. Cell Biol..

[90] King L, Plun-Favreau H (2017). Mitophagy. Park. Dis. Mol. Mech. Underlying Pathol..

[91] Ogasawara M (2016). The protective role of protein L-isoaspartyl (D-aspartate) O-methyltransferase for maintenance of mitochondrial morphology in A549 cell. Exp. Lung Res..

[92] Park JH (2020). Alpha-synuclein-induced mitochondrial dysfunction is mediated via a sirtuin 3-dependent pathway. Mol. Neurodegener..

[93] Han F (2019). Neuroinflammation and myelin status in Alzheimer’s disease, Parkinson’s disease, and normal aging brains: A small sample study. Park. Dis..

[94] Lasagna-Reeves CA (2011). Tau oligomers impair memory and induce synaptic and mitochondrial dysfunction in wild-type mice. Mol. Neurodegener..

[95] Devi L, Raghavendran V, Prabhu BM, Avadhani NG, Anandatheerthavarada HK (2008). Mitochondrial import and accumulation of alpha-synuclein impair complex I in human dopaminergic neuronal cultures and Parkinson disease brain. J. Biol. Chem..

[96] Ballard PA, Tetrud JW, Langston JW (1985). Permanent human parkinsonism due to 1-methyl-4-phenyl-1,2,3,6-tetrahydropyridine (MPTP): Seven cases. Neurology.

[97] Nicklas WJ, Youngster SK, Kindt MV, Heikkila REIV (1987). MPTP, MPP+ and mitochondrial function. Life Sci..

[98] Lickteig B, Wimalasena VK, Wimalasena K (2019). N-Methyl-4-phenylpyridinium scaffold-containing lipophilic compounds are potent complex I inhibitors and selective dopaminergic toxins. ACS Chem. Neurosci..

[99] Murphy D, Patel H, Wimalasena K (2021). Caenorhabditis elegans model studies show MPP+is a simple member of a large group of related potent dopaminergic toxins. Chem. Res. Toxicol..

[100] Ge P, Dawson VL, Dawson TM (2020). PINK1 and Parkin mitochondrial quality control: a source of regional vulnerability in Parkinson’s disease. Mol. Neurodegener..

[101] Mendonça CF (2019). Proteomic signatures of brain regions affected by tau pathology in early and late stages of Alzheimer’s disease. Neurobiol. Dis..

[102] Burman JL, Yu S, Poole AC, Decal RB, Pallanck L (2012). Analysis of neural subtypes reveals selective mitochondrial dysfunction in dopaminergic neurons from parkin mutants. Proc. Natl. Acad. Sci. U. S. A..

[103] McWilliams TG (2018). Basal mitophagy occurs independently of PINK1 in mouse tissues of high metabolic demand. Cell Metab..

[104] Oshima Y (2021). Parkin-independent mitophagy via Drp1-mediated outer membrane severing and inner membrane ubiquitination. J. Cell Biol..

[105] Xian H, Yang Q, Xiao L, Shen HM, Liou YC (2019). STX17 dynamically regulated by Fis1 induces mitophagy via hierarchical macroautophagic mechanism. Nat. Commun..

[106] Antony PMA, Diederich NJ, Balling R (2011). Parkinson’s disease mouse models in translational research. Mamm. Genome.

[107] Kageyama Y (2014). Parkin-independent mitophagy requires Drp1 and maintains the integrity of mammalian heart and brain. EMBO J..

[108] Mandal A, Drerup CM (2019). Axonal transport and mitochondrial function in neurons. Front. Cell. Neurosci..

[109] Guillaud L, El-Agamy SE, Otsuki M, Terenzio M (2020). Anterograde axonal transport in neuronal homeostasis and disease. Front. Mol. Neurosci..

[110] Wan, W. *et al.* Regulation of mitophagy by sirtuin family proteins: A vital role in aging and age-related diseases. *Front. Aging Neurosci.* **0**, 469 (2022). 10.3389/fnagi.2022.845330 PMC9124796 35615591

[111] Verdin E, Hirschey MD, Finley LWS, Haigis MC (2010). Sirtuin regulation of mitochondria - Energy production, apoptosis, and signaling. Trends Biochem. Sci..

[112] Nagashima S (2019). MITOL deletion in the brain impairs mitochondrial structure and ER tethering leading to oxidative stress. Life Sci. Alliance.

[113] Perez-Riverol Y (2022). The PRIDE database resources in 2022: A hub for mass spectrometry-based proteomics evidences. Nucleic Acids Res..

