# Supplementary figures and images for: Longitudinal in vivo metabolic labeling reveals tissue-specific mitochondrial proteome turnover rates and proteins selectively altered by parkin deficiency
Source: Sci Rep. 2023 Jul 14;13:11414. doi: 10.1038/s41598-023-38484-0 (PMC10349111; doi:10.1038/s41598-023-38484-0)

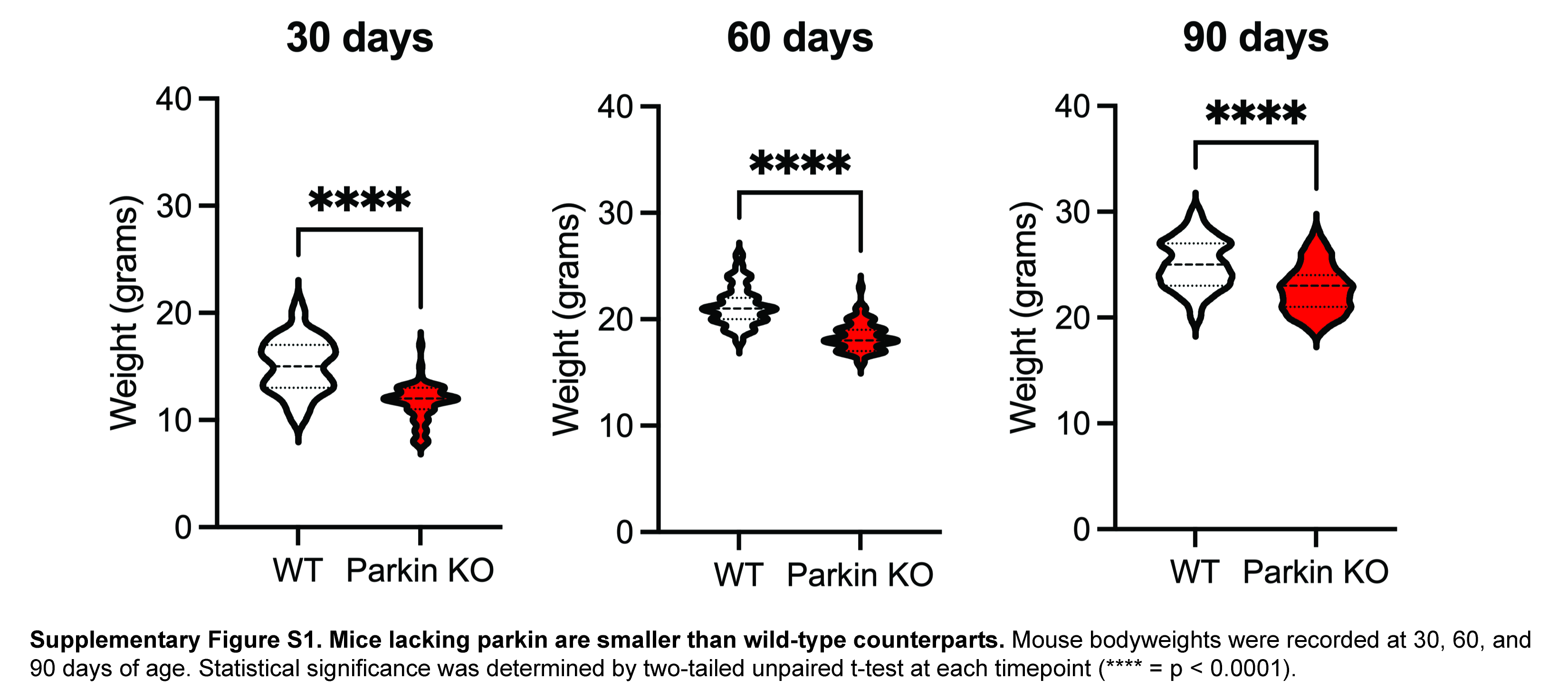

Supplement: Supplementary file 1 — Supplementary Figure 1. [file 41598_2023_38484_MOESM1_ESM.tif]

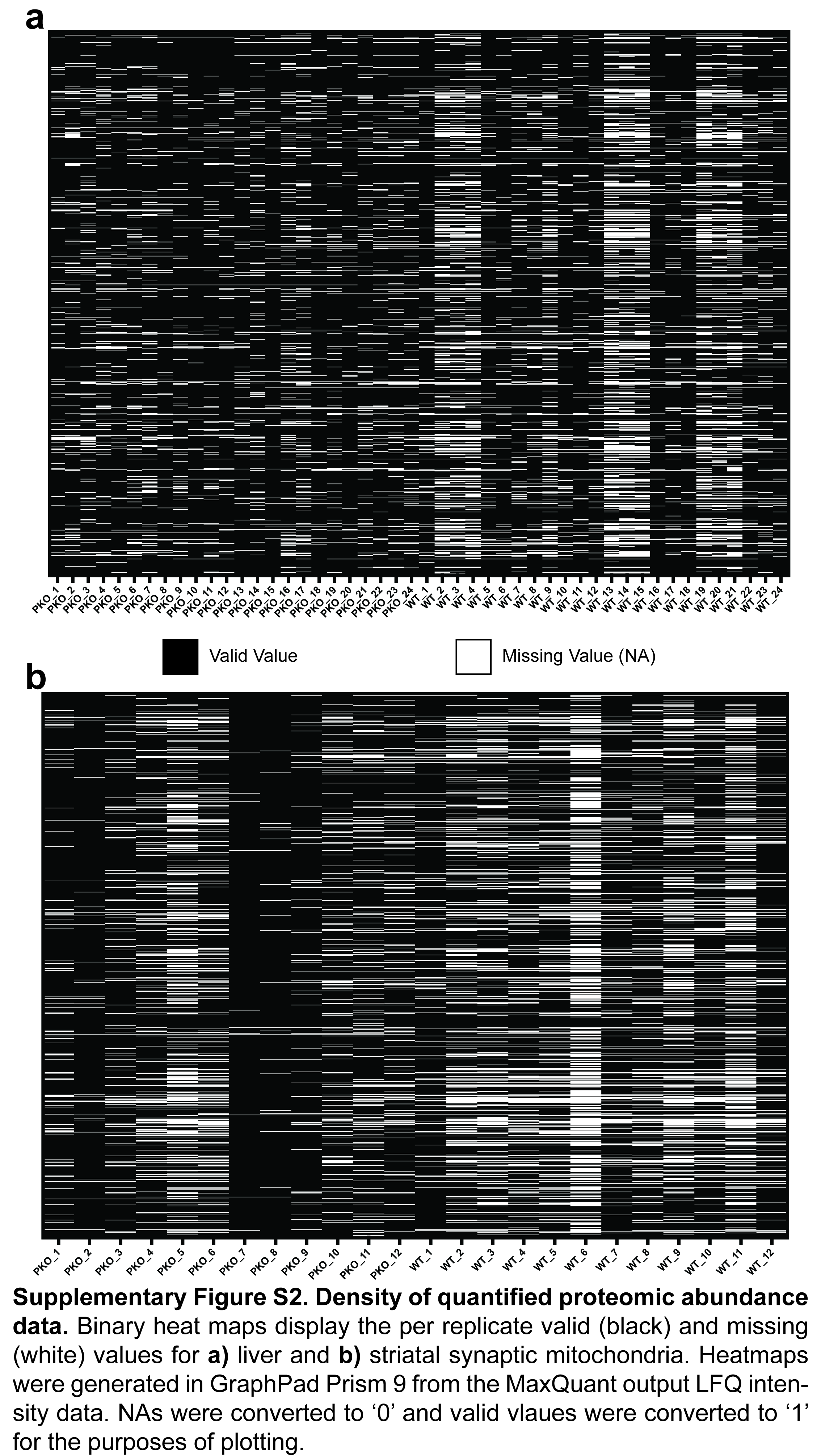

Supplement: Supplementary file 2 — Supplementary Figure 2. [file 41598_2023_38484_MOESM2_ESM.tif]

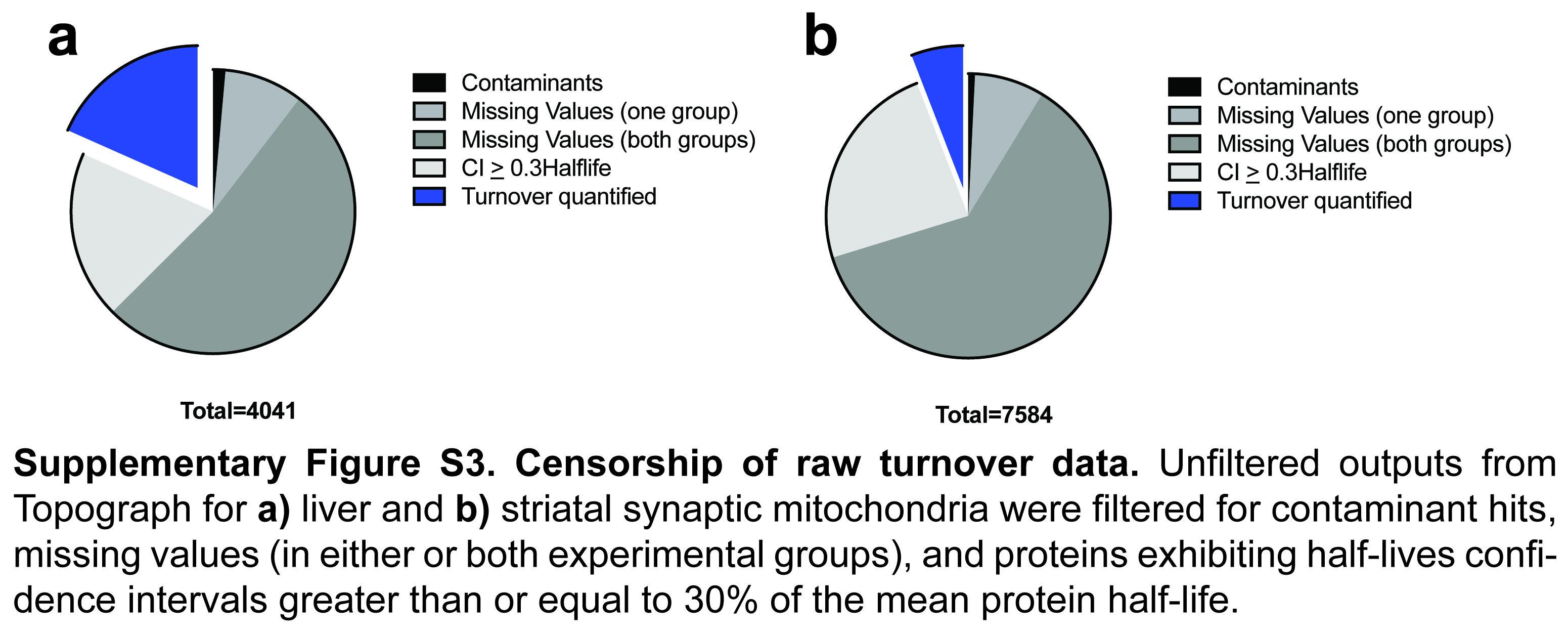

Supplement: Supplementary file 3 — Supplementary Figure 3. [file 41598_2023_38484_MOESM3_ESM.tif]

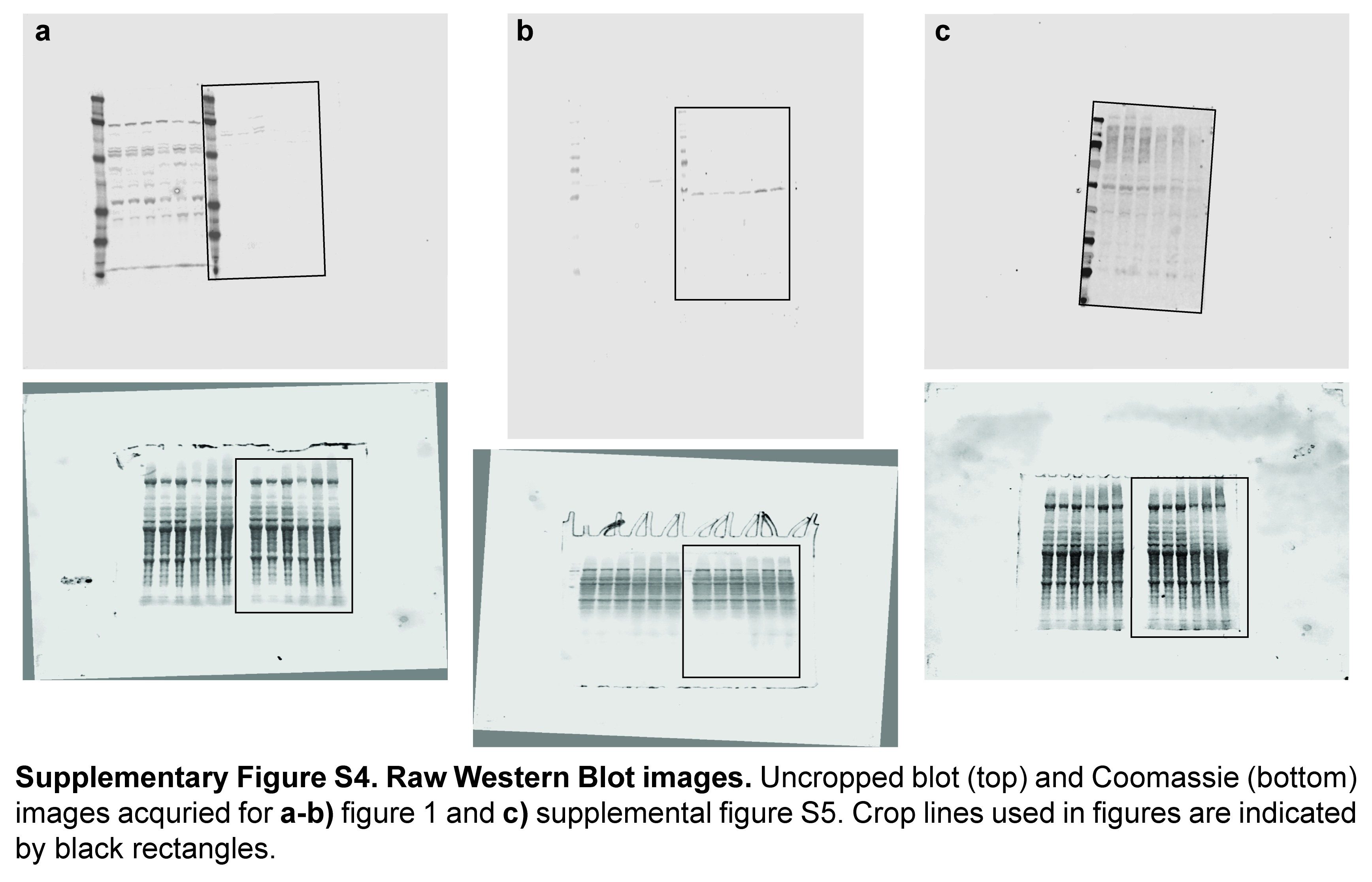

Supplement: Supplementary file 4 — Supplementary Figure 4. [file 41598_2023_38484_MOESM4_ESM.tif]

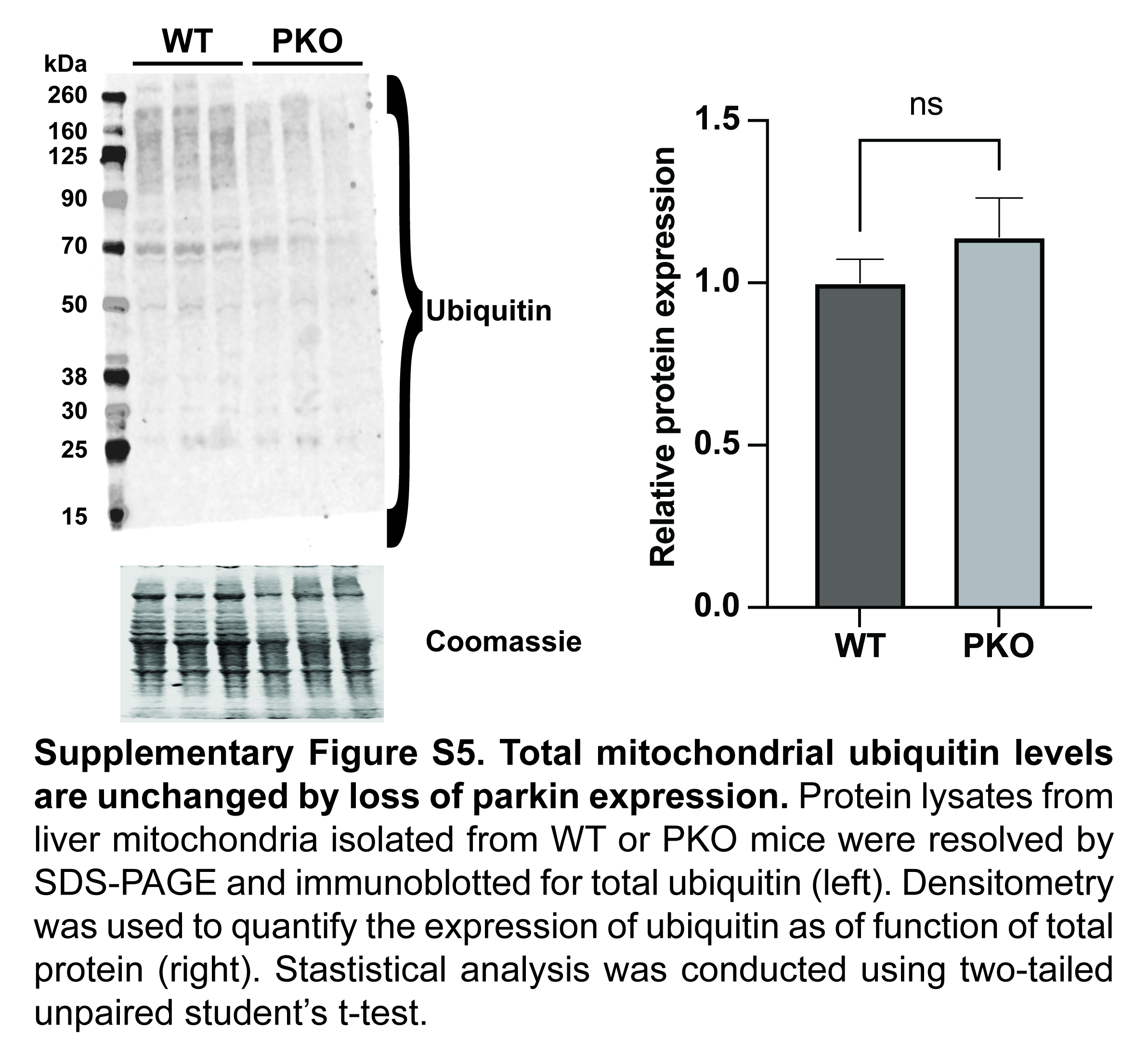

Supplement: Supplementary file 5 — Supplementary Figure 5. [file 41598_2023_38484_MOESM5_ESM.tif]
